# Thermal conductivity of glasses: first-principles theory and applications

**DOI:** 10.1038/s41524-023-01033-4

**Published:** 2023-06-19

**Authors:** Michele Simoncelli, Francesco Mauri, Nicola Marzari

**Affiliations:** 1 Theory of Condensed Matter Group of the Cavendish Laboratory, University of Cambridge, Cambridge, UK; 2 Dipartimento di Fisica, Università di Roma La Sapienza, Roma, Italy; 3 Theory and Simulation of Materials (THEOS) and National Centre for Computational Design and Discovery of Novel Materials (MARVEL), École Polytechnique Fédérale de Lausanne, Lausanne, Switzerland

**Keywords:** Computational methods, Condensed-matter physics

## Abstract

Predicting the thermal conductivity of glasses from first principles has hitherto been a very complex problem. The established Allen-Feldman and Green-Kubo approaches employ approximations with limited validity—the former neglects anharmonicity, the latter misses the quantum Bose-Einstein statistics of vibrations—and require atomistic models that are very challenging for first-principles methods. Here, we present a protocol to determine from first principles the thermal conductivity *κ*(*T*) of glasses above the plateau (i.e., above the temperature-independent region appearing almost without exceptions in the *κ*(*T*) of all glasses at cryogenic temperatures). The protocol combines the Wigner formulation of thermal transport with convergence-acceleration techniques, and accounts comprehensively for the effects of structural disorder, anharmonicity, and Bose-Einstein statistics. We validate this approach in vitreous silica, showing that models containing less than 200 atoms can already reproduce *κ*(*T*) in the macroscopic limit. We discuss the effects of anharmonicity and the mechanisms determining the trend of *κ*(*T*) at high temperature, reproducing experiments at temperatures where radiative effects remain negligible.

## Introduction

The thermal conductivity of glasses is a key property for many and diverse technological applications, including, e.g., the microelectronics^1^, pharmaceutical^2^, aerospace^3,4^, optics^5^, and construction^6^ industries. To a large degree of universality, for glasses there are three characteristic intervals in the temperature dependence of the thermal conductivity *κ*(*T*)^7–10^: (i) the low temperature region (*T* ≲ 1 *K*) where *κ*(*T*) ~ *T*^2^; (ii) the intermediate temperature region (5 ≲ *T* ≲ 25 *K*) where the conductivity displays a plateau ($\kappa (T) \sim {{{\rm{constant}}}}$); (iii) the above-the-plateau region (*T* ≳ 30 K) where the thermal conductivity increases with temperature. The trend observed in region (i) is usually explained relying on the phenomenological^7,11–13^ ‘tunneling two-level system’ (TLS) model^14,15^. The plateau found in the region (ii) has motivated several studies, which explored its possible connection with the boson peak^16–19^. The above-the-plateau region (iii)—which is the most relevant one for the aforementioned technological sectors—has up to now been investigated using the Allen-Feldman (AF) formulation^8,9,20^ or various molecular-dynamics (MD) methods. Specifically, the various MD-based methods employed in past works are: (i) the Green-Kubo formulation in combination with classical^21–24^, first-principles^25,26^, or machine learning^27–30^ MD simulations (GKMD); (ii) the approach-to-equilibrium MD (AEMD) in combination with classical potentials^31^ or first-principles molecular dynamics^32–34^; (iii) non-equilibrium MD simulations (NEMD) in combination with classical^21,35,36^ or machine-learned^37^ potentials. Past works relying on these formulations have highlighted two major challenges. First, achieving computational convergence with respect to supercell sizes and simulations times is onerous and problematic^20,21,38^, leading to the conclusion that accurate calculations require models containing thousands of atoms, and simulation times of the order of tens to hundreds of picoseconds; this yields a computational cost that is prohibitively high for direct first-principles calculations. Second, neither Allen-Feldman nor MD formulations can immediately ensure a comprehensive description of thermal transport, since the former neglects anharmonicity (thus it is in principle accurate only in the low-temperature regime where anharmonicity phases out^9^), and the latter, while accounting for anharmonicity exactly, misses the quantum Bose-Einstein statistics of atomic vibrations^39^, relevant at low temperatures.

The recently developed Wigner formulation^40,41^ has shown that the two established microscopic heat-conduction mechanisms for crystals and glasses—i.e., the particle-like propagation of phonon wavepackets described by the Peierls-Boltzmann equation^42,43^, and the wave-like tunneling mechanisms described by the AF equation^8,9^, respectively—both emerge as limiting cases from a unified transport equation. We note, in passing, that here the ‘diffuson’ mechanisms described by Allen-Feldman theory is denoted with ‘wave-like tunneling’ because it bears some analogies to the electronic Zener interband tunneling^44^, more details about the terminology can be found in refs. ^40,41^. The Wigner formulation offers a comprehensive approach to describe heat transport in solids, encompassing: (i) ‘simple crystals’, characterized by phonon interband spacings much larger than the linewidths, where particle-like propagation dominates and the Peierls-Boltzmann limit is accurate; (ii) glasses, where wave-like tunneling is relevant and the Wigner formulation extends AF theory accounting for anharmonicity; (iii) the intermediate regime of ‘complex crystals’, where particle-like propagation and wave-like tunneling are simultaneously present and neither Peierls-Boltzmann or Allen-Feldman are valid. This intermediate regime is common and prevalent in crystals characterized by phonon interband spacings smaller than the linewidths and featuring ultralow thermal conductivity (e.g., those used in thermoelectrics^45^ or thermal barrier coatings^41^). The Wigner formulation for transport has paved the way, as we will see, to tackle the aforementioned challenges of comprehensively describing transport in glasses accounting for the interplay between Bose-Einstein statistics, anharmonicity, and disorder. It is worth mentioning that recent works^38,46^ employed a comprehensive ‘quasi-harmonic Green-Kubo’ transport framework—formally equivalent to the Wigner formulation^47,48^—in combination with classical potentials to describe heat transport in amorphous materials. Still, the challenge of combining the Wigner formulation with first-principles methods remains, due to a structural complexity larger than simple crystals^49^, disordered crystals^50,51^, and complex crystals, for which several advances have recently been made^40,41,45,52–58^.

Here, we present a protocol that addresses simultaneously both the aforementioned challenges, enabling accurate first-principles predictions for the thermal conductivity of glasses, and combining the Wigner transport equation^40,41^ (WTE) with convergence-acceleration techniques; it is first discussed in the limiting case of vanishing anharmonicity, where the WTE conductivity reduces to the harmonic AF^8,9^ conductivity, and then extended to account for the effects of anharmonicity and evaluate from finite-size models of glasses the bulk limit of the anharmonic WTE conductivity. We showcase the protocol in vitreous silica (*v*-SiO_2_)—a paradigmatic glass that is employed in many and diverse technologies^1–6^—extending the simulations to very large cell sizes thanks to the use of machine-learned potentials (the recently developed Gaussian Approximation Potential^59^ (GAP) for silica polymorphs^60^). The GAP potential we employ has been generated from first-principles calculations performed using the SCAN functional^61^, and yields quantum-accurate predictions for the vibrational properties of various silica polymorphs^60^. Here, we employ GAP to describe the vibrational properties and evaluate the thermal conductivity—both in the harmonic Allen-Feldman or in the anharmonic Wigner framework—of models of *v*-SiO_2_ having very different sizes, showing how our protocol allows to accurately evaluate the bulk limit of the harmonic or anharmonic conductivity using models containing less than 200 atoms, that are in very good agreement with the macroscopic cell limit of 5184 atoms^60^.

After having validated the protocol, we employ it to study the conductivity of *v*-SiO_2_ fully from first principles using models of *v*-SiO_2_ containing 108^62^, 144^63,64^, and 192 atoms^65^. We discuss how the AF or WTE conductivities change if the widely used semi-empirical van Beest-Kramer-van Santen^66,67^ (BKS) potential, or the state-of-the-art GAP potential for silica polymorphs^60^, are used in place of first-principles calculations to compute the vibrational properties of *v*-SiO_2_. We elucidate analogies and differences between the harmonic and the anharmonic conductivity, relying on the latter to show how the high-temperature trend of *κ*(*T*) is determined by how the velocity-operator elements (whose values regulate the amount of heat transferred by wave-like tunneling between vibrational eigenstates) vary when the energy difference between coupled eigenstates increases. Finally, we rationalize these results at the microscopic level, relying on Allen-Feldman’s harmonic diffusivity and extending such quantity to account for anharmonicity, but also showing that anharmonicity has negligible effects on the conductivity and diffusivity of *v*-SiO_2_.

### Wigner formulation of thermal transport

As anticipated, the WTE formalism is general and can be used to describe solids with variable degrees of disorder, ranging from ordered crystals with small primitive cells to disordered glasses with diverging primitive cell (in this latter case, for sufficiently large primitive cells periodic-boundary conditions become irrelevant and the Brillouin zone (BZ) reduces to the point **q** = **0** only). In practice, in numerical simulations non-periodic glasses can be approximately described as crystals with large but finite primitive cells, thus having a small but finite BZ that includes wavevectors different from **q** = **0**. We will discuss in the next section the conditions under which non-periodic glasses can be simulated in periodic-boundary conditions, after having introduced here the salient features of the WTE formulation in the general case where vibrations can have a wavevector different from **q** = **0**.

For systems with low conductivity, which are the focus of this work, the WTE can be solved accurately within the so-called single-mode relaxation-time approximation (SMA, see Methods for details), which allows to write the conductivity in the following compact form:

1 $$\begin{array}{lll}\kappa \,=\,\frac{1}{{{{\mathcal{V}}}}{N}_{{{{\rm{c}}}}}}\mathop{\sum}\limits_{{{{\bf{q}}}},s,{s}^{{\prime} }}\frac{\omega {({{{\bf{q}}}})}_{s}+\omega {({{{\bf{q}}}})}_{{s}^{{\prime} }}}{4}\left(\frac{C{({{{\bf{q}}}})}_{s}}{\omega {({{{\bf{q}}}})}_{s}}+\frac{C{({{{\bf{q}}}})}_{{s}^{{\prime} }}}{\omega {({{{\bf{q}}}})}_{{s}^{{\prime} }}}\right)\frac{\parallel {\mathbf{v}}{({{{\bf{q}}}})}_{s,{s}^{{\prime} }}{\parallel }^{2}}{3}\\ \qquad\times \,\pi {{{{\mathcal{F}}}}}_{[\Gamma {({{{\bf{q}}}})}_{s}+\Gamma {({{{\bf{q}}}})}_{{s}^{{\prime} }}]}(\omega {({{{\bf{q}}}})}_{s}-\omega {({{{\bf{q}}}})}_{{s}^{{\prime} }}),\end{array}$$

where the wavevector **q** and the mode index *s* label a vibrational eigenstate having energy ℏ*ω*(**q**)_*s*_, anharmonic linewidth ℏΓ(**q**)_*s*_, and specific heat

2 $$C{({{{\bf{q}}}})}_{s}=C[\omega {({{{\bf{q}}}})}_{s}]=\frac{{\hslash }^{2}{\omega }^{2}{({{{\bf{q}}}})}_{s}}{{k}_{{{{\rm{B}}}}}{T}^{2}}{\mathsf{N}}{({{{\bf{q}}}})}_{s}\left({\mathsf{N}}{({{{\bf{q}}}})}_{s}+1\right)$$

(${\mathsf{N}}{({{{\bf{q}}}})}_{s}={[\exp (\hslash \omega {({{{\bf{q}}}})}_{s}/{k}_{{{{\rm{B}}}}}T)-1]}^{-1}$ is the Bose-Einstein distribution at temperature *T*); the quantity

3 $$\parallel {{\mathbf{v}}}{({{{\bf{q}}}})}_{s,{s}^{{\prime} }}{\parallel }^{2}=\mathop{\sum }\limits_{\alpha =1}^{3}{{\mathsf{v}}}^{\alpha }{({{{\bf{q}}}})}_{s,{s}^{{\prime} }}{{\mathsf{v}}}^{\alpha }{({{{\bf{q}}}})}_{{s}^{{\prime} },s}$$

denotes the square modulus of the velocity operator^41^ between eigenstates *s* and ${s}^{{\prime} }$ at the same wavevector **q** (*α* in these expressions denotes a Cartesian direction, and since vitreous solids are in general isotropic, the scalar conductivity (1) is computed as the average trace of the tensor *κ*^*αβ*^ given by Eq. (47) of ref. ^41^; *N*_c_ is the number of **q**-points entering in such a summation and ${{{\mathcal{V}}}}$ is the primitive-cell volume). Finally, ${{{\mathcal{F}}}}$ is a Lorentzian distribution having a full width at half maximum (FWHM) equal to $\Gamma {({{{\bf{q}}}})}_{s}+\Gamma {({{{\bf{q}}}})}_{{s}^{{\prime} }}$:

4 $${{{{\mathcal{F}}}}}_{[\Gamma {({{{\bf{q}}}})}_{s}+\Gamma {({{{\bf{q}}}})}_{{s}^{{\prime} }}]}\left(\omega {({{{\bf{q}}}})}_{s}-\omega {({{{\bf{q}}}})}_{{s}^{{\prime} }}\right)=\frac{1}{\pi }\frac{\frac{1}{2}\left(\Gamma {({{{\bf{q}}}})}_{s}+\Gamma {({{{\bf{q}}}})}_{{s}^{{\prime} }}\right)}{{\left(\omega {({{{\bf{q}}}})}_{s}-\omega {({{{\bf{q}}}})}_{{s}^{{\prime} }}\right)}^{2}+\frac{1}{4}{\left(\Gamma {({{{\bf{q}}}})}_{s}+\Gamma {({{{\bf{q}}}})}_{{s}^{{\prime} }}\right)}^{2}}.$$

In a crystal the primitive cell and the BZ have a finite volume and can be univocally chosen relying on crystallographic conditions^68^; wavevectors are good quantum numbers and phonon group velocities are well defined. Under these circumstances, it is useful to rewrite the WTE conductivity (1) as sum of two terms, *κ* = *κ*_*P*_ + *κ*_*C*_; specifically, the term *κ*_*P*_ (referred to as ‘populations conductivity’) is determined by the diagonal ($s={s}^{{\prime} }$) or perfectly degenerate ($s\,\ne\, {s}^{{\prime} }$ with $\omega {({{{\bf{q}}}})}_{s}=\omega {({{{\bf{q}}}})}_{{s}^{{\prime} }}$) terms^41^ in the summation in the conductivity (1); in crystals such a term describes the Peierls-Boltzmann particle-like heat conduction (averaged over the spatial directions), since the average trace of the Peierls-Boltzmann conductivity tensor can be written as ${\kappa }_{P}=\frac{1}{3}{\sum }_{\alpha }{\kappa }_{P}^{\alpha \alpha }$ with ${\kappa }_{P}^{\alpha \alpha }=\frac{1}{{{{\mathcal{V}}}}{N}_{C}}{\sum }_{{{{\bf{q}}}}s}C[\omega {({{{\bf{q}}}})}_{s}]{{\mathsf{v}}}^{\alpha }{({{{\bf{q}}}})}_{s,s}{\Lambda }^{\alpha }{({{{\bf{q}}}})}_{s}$, i.e., as particle-like vibrations having absolute energy ℏ*ω*(**q**)_*s*_ (thus specific heat *C*[*ω*(**q**)_*s*_]) and propagating between collisions over a length ${\Lambda }^{\alpha }{({{{\bf{q}}}})}_{s}={{\mathsf{v}}}^{\alpha }{({{{\bf{q}}}})}_{s,s}{[\Gamma {({{{\bf{q}}}})}_{s}]}^{-1}$. We note, in passing, that here we have exploited the possibility to diagonalize the velocity operator in the degenerate subspace^41,69^. Conversely, non-degenerate off-diagonal elements (‘coherences’^40,41^) account for a different ‘Wigner’ conduction mechanisms: they do not have an absolute energy akin to that of a particle-like excitation, but are characterized by an energy difference $\hslash \omega {({{{\bf{q}}}})}_{s}-\hslash \omega {({{{\bf{q}}}})}_{{s}^{{\prime} }}$ and describe a wave-like tunneling conduction mechanisms akin to the electronic Zener interband tunnelling^44^. It has been shown in refs. ^40,41,70^ that in simple crystals particle-like mechanisms dominate and thus *κ*_P_ ≫ *κ*_C_, while in complex crystals both these mechanisms are relevant, and *κ*_P_ and *κ*_C_ are of the same order. Finally, refs. ^40,41^ have shown that in the ordered limit describing a harmonic glass^8,9^, Eq. (1) reduces to the AF formula for the conductivity of glasses. Specifically, such an ordered limit requires first to describe a (structurally stable^71–78^) glass as the limiting case of a disordered but periodic crystal with an increasingly larger primitive cell (i.e.,${{{\mathcal{V}}}}\to \infty$ and thus with the BZ reducing to the point **q** = **0** only^8,9^), and then letting each linewidth go to the same infinitesimal broadening ℏη^8,9^, ℏΓ(**q**)_*s*_ → ℏ*η* → 0, ∀ *s* and **q** = **0**. Under these ideal circumstances only **q** = **0** is considered in the sum in Eq. (1), and the Lorentzian distribution (4) becomes a Dirac *δ*,

5 $$\mathop{\lim }\limits_{\eta \to 0}\left[\mathop{\lim }\limits_{{{{\mathcal{V}}}}\to \infty }{{{{\mathcal{F}}}}}_{[2\eta ]}(\omega {({{{\bf{q}}}})}_{s}-\omega {({{{\bf{q}}}})}_{{s}^{{\prime} }})\right]=\delta \left(\omega {({{{\bf{q}}}})}_{s}-\omega {({{{\bf{q}}}})}_{{s}^{{\prime} }}\right),$$

implying that the WTE conductivity (1) reduces exactly to the AF formula for the conductivity of glasses (Eq. (3) of ref. ^8^). In practice, this ideal bulk-glass limit cannot be reached in numerical calculations, and anharmonic linewidths strongly vary with temperature. In the next sections we first discuss a protocol that allows to accurately describe glasses at the AF harmonic level using finite-size models, and then we rely on the WTE to extend such a protocol accounting for anharmonicity.

## Results

### Protocol to evaluate the harmonic Allen-Feldman conductivity with finite-size models

From a microscopic viewpoint, AF conductivity (Eq. (1) and (5) with **q** = **0** only) describes heat transport in an ideal bulk glass as being mediated by a transfer of energy between vibrations that are degenerate in energy (see Eq. (5)) and not localized in the Anderson sense^79^ (this requirement is needed to have non-zero velocity operator elements in Eq. (1)). In actual calculations a glass is approximately described as a crystal having a primitive cell containing a large but finite number of atoms *N*_at_. Such an approximation has two important implications. First, the BZ corresponding to the (large) finite-size model does not reduce to **q** = **0** only but has a (small) finite volume. Second, in a realistic finite-size model of a glass perfectly degenerate vibrational modes are supposed to be absent (this because exact degeneracies are due to point-group symmetries^80^, which are not present in amorphous systems); thus, the vibrational spectrum is characterized by an average spacing between vibrational energy levels equal to

6 $$\hslash \Delta {\omega }_{{{{\rm{avg}}}}}=\frac{\hslash {\omega }_{\max }}{3{N}_{{{{\rm{at}}}}}},$$

where $\hslash {\omega }_{\max }$ is the maximum vibrational energy of the solid (which strongly depends on the chemical composition and negligibly depends on disorder, see Fig. 10 in Methods) and 3*N*_at_ is the number of vibrational modes at a fixed **q** point. Eq. (6) underlines how degenerate vibrational modes determining the harmonic conductivity emerge as a consequence of disorder, since increasing the accuracy in the description of disorder (i.e., increasing *N*_at_) yields a decrease of the average energy-level spacing, implying that in the ideal limit of a bulk glass (*N*_at_ → *∞*) adjacent vibrational eigenstates become degenerate (hereafter we will use the term ‘quasi-degenerate’ to refer to these adjacent vibrational eigenstates that become degenerate in the ideal limit of a bulk glass).

These two properties offer important insights on how to evaluate the strength of finite-size effects in glasses and consequently extrapolate from the finite-size ‘reference cell’ of the model the behavior of the ideal (infinite) glass. Specifically, the presence of a BZ with a small but finite volume implies that Fourier interpolations can be used to sample vibrations in a *n* × *n* × *n* supercell of the finite periodic model. This procedure simulates a system where vibrations are commensurate to a system that is *n* × *n* × *n* times larger than the reference cell used, but where the disorder length scale remains limited by the size of the reference cell. Within this scheme one can obtain information about finite-size effects in multiple ways: (i) for a given finite model, one can study the differences between a calculation performed at **q** = **0** only, and a calculation on a *n* × *n* × *n* **q** mesh; (ii) one can repeat the analysis at the previous point employing models having larger and larger reference cells—for sufficiently large models one expects to achieve computational convergence, with the calculation at **q** = **0** only and that on the **q** mesh yielding indistinguishable results. Moreover, in order to retain the key physical property that couplings between quasi-degenerate eigenstates can occur and contribute to heat transport, the Dirac *δ* (Eq. (5)) needs to be replaced with a smooth distribution having a broadening *η* of the order of the average energy-level spacing^8,9,20^ ℏΔ*ω*_avg_; otherwise, no couplings would take place in any system represented by a finite-size periodic supercell. Within such a numerical scheme *η* is just a computational parameter, and as such one expects to find a range of values for *η* (the domain of convergence) for which the conductivity is independent from *η*. The considerations above show that the calculation of the harmonic AF conductivity in finite-size models bears some procedural analogies to electronic-structure calculations in metals, where the BZ is integrated using a discrete mesh and the Dirac *δ* identifying the Fermi level is broadened with an aptly chosen smearing function^81–83^, and one needs to identify a range of ‘converged values’ for the smearing and for the size of the BZ-integration mesh for which quantities such as the total energy are practically independent from the smearing.

**Fig. 1:**
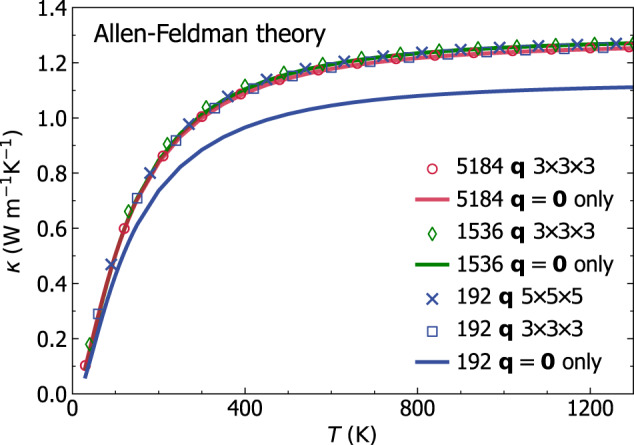
Convergence of the Allen-Feldman theory in finite-size *v*-SiO_2_ models. The AF conductivities for a 192-, 1536-, and 5184-atom model are in blue, green, and red, respectively; calculations at **q** = **0** only are solid lines, calculations using the **q** interpolation on a 3 × 3 × 3 (5 × 5 × 5) mesh are empty symbols (crosses). All calculations employ a Gaussian broadening *η* = 4 cm^−1^ for the Dirac *δ* (Eq. (5), corresponding to a FWHM larger than the average energy-level spacing of these models, see text and Fig. 2). The discrepancy between the solid blue line and all the other data shows that the **q** interpolation is necessary to achieve convergence using a 192-atom model; the green and red lines show that convergence is achieved already with a calculation at **q** = **0** in both the 1536- and 5184-atom models.

**Fig. 2:**
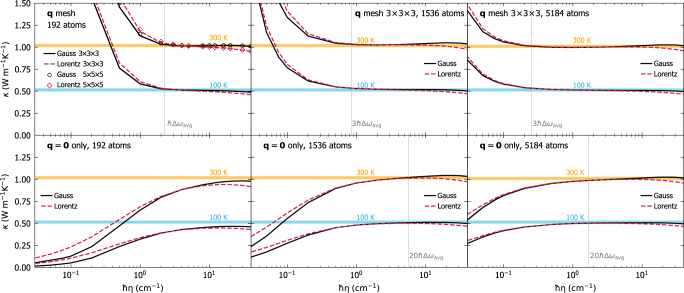
Convergence of the AF conductivity for *v* − SiO_2_ with respect to the broadening *η* for the Dirac *δ*, for a 192-atom model (left), for a 1536-atom model (center), and for a 5184-atom model (right). Every dashed-red line is a Lorentzian having FWHM 2*η*, every solid-black line is a Gaussian with variance *η*^2^*π*/2 (both distributions have the same maximum (*πη*)^−1^). Top row: evaluating the AF conductivity using the **q** interpolation and the Gaussian broadening yields a `convergence plateau', i.e., range of value for *η* for which the conductivity is not sensitive to the value of *η* (orange and blue lines show the converged `bulk' value for the AF conductivity at 100 and 300 K, respectively). The Gaussian yields a wider and more clear convergence plateau compared to the Lorentzian. Bottom row, calculations of the AF conductivity at **q** = **0**: the small 192-atom model underestimates the bulk limit; in contrast, the medium 1536-atom model and the large 5184-atom model yield a convergence plateau also at **q** = **0**, with the largest model featuring the widest convergence plateau. We note that the plateaus at **q** = **0** for the 1536- and the 5184-atom models are narrower than the corresponding ones obtained using the **q** interpolation. The vertical dotted lines are indicative of the minimum broadening for which computational convergence is achieved. The opposite trend of the broadening-conductivity curve obtained using the **q** mesh or **q** = **0** is discussed in Sec. *Extension of the protocol to evaluate the anharmonic Wigner conductivity*.

Pioneering work^8–10,20^ evaluated the AF conductivity broadening the Dirac *δ* (Eq. (5)) with a fat-tailed Lorentzian distribution having FWHM of the order of ℏΔ*ω*_avg_, limited calculations to **q** = **0** to reduce the computational cost, and relied on smoothness arguments^9^ to extrapolate the bulk limit from finite-size models. Nevertheless, recent work^21,38,84^ have highlighted challenges in achieving computational convergence following such computational protocol. Therefore, here we take inspiration from the computational techniques alluded to above for electronic-structure calculations to tackle the problem of achieving convergence in the calculation of the AF conductivity, using vitreous silica as a paradigmatic test case. We first show in Fig. 1 that state-of-the-art models of vitreous silica^60^ (*v*-SiO_2_) containing 192, 1536, or 5184 atoms (all studied using a recently developed GAP potential^60^, see Methods for details) yield bulk AF conductivities that are perfectly compatible, provided these are computed using a light-tailed Gaussian broadening having a FWHM larger than the average energy-level spacing (more details on the convergence domain for the broadening parameter are discussed later), and a Fourier interpolation is used to extrapolate the bulk limit from the small 192-atom model (we note that the Fourier interpolation has a negligible effect on the 1536-atom and 5184-atom models, confirming the aforementioned expectations). The details on the domain of convergence for the broadening parameter *η* and mesh used for the BZ sampling are reported in Fig. 2, where we show that computational convergence can be achieved for atomistic models containing 192, 1536, and 5184 atoms (i.e., there exists a range of values for *η*—the ‘convergence plateau’—over which the conductivity is not sensitive to the value of *η*). As the size of the reference cell of the atomistic model increases, the convergence plateau extends to smaller values of *η*; this in agreement with the expectation, based on Eq. (6), that larger models allow for more accurate approximations of the Dirac *δ*. Importantly, we also show that the Gaussian broadening yields a wider convergence plateau compared to the Lorentzian broadening, especially at low temperatures where anharmonicity phases out and thus the harmonic AF theory is accurate. We note in passing that the improved computational performance found here for the Gaussian broadening compared to the Lorentzian broadening bears analogies to density-functional-theory calculations for metals, where refined representations of the Dirac delta^81–83,83,85^ are used in place of the Fermi-Dirac thermal broadening^86^ to improve convergence with respect to Brillouin-zone sampling. The analysis of Fig. 2 demonstrates that the evaluation of the AF conductivity at **q** = **0** only does not show clear convergence with respect to the broadening for the 192-atom model; a convergence plateau emerges instead if the Fourier interpolation is employed to extrapolate to the bulk limit. In contrast, in the 1536- and 5184-atom models computing the AF conductivity at **q** = **0** only or using an interpolation mesh does not produce significant differences, provided a value of *η* belonging to the convergence plateau is used. This shows that the protocol of using the **q** mesh interpolation and (ideally) Gaussian broadening to compute the AF conductivity allows to achieve computational convergence in three small models (containing ≲ 200 atoms) typically affordable in first-principles studies; in *v*-SiO_2_ models containing more than 1500 atoms are accurate already at **q** = **0**, and allow to determine a convergence plateau for the broadening *η*.

### Extension of the protocol to evaluate the anharmonic Wigner conductivity

**Fig. 3:**
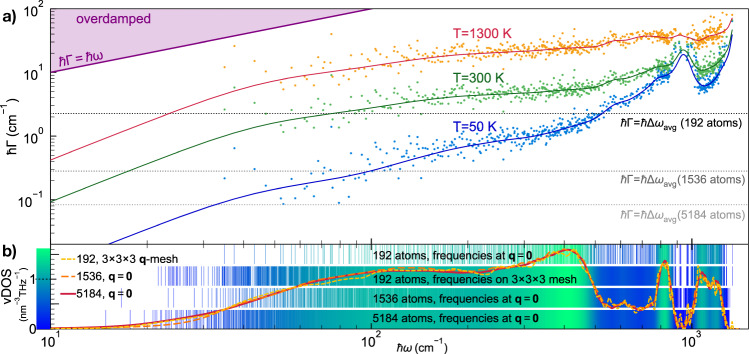
Vibrational frequencies and anharmonic linewidths of *v*-SiO_2_. **a** The scatter points are the linewidths as a function of frequency and temperature for the 192-atom model; these are computed at **q** = **0** only accounting for third-order anharmonicity^87–91^ and natural-abundance isotopic mass disorder^92^. The solid lines represent a coarse graining of the frequency-linewidth distribution into a single-valued function^51,93^ Γ_a_[*ω*] (see Methods). The horizontal lines show the average energy-level spacing for the 192-atom (black), 1536-atom (dark gray), and 5184-atom (light gray) models; for each model all the vibrations with linewidth around and below the corresponding average energy-level spacing line are not accurately accounted for by the bare WTE (Eq. (1) and (4)), and need to be regularized (see text). **b** The solid red line is the vibrational density of state (vDOS) of the 5184-atom model computed at **q** = **0**. The dashed yellow (orange) line is the vDOS of the 192-atom model computed using a 3 × 3 × 3 **q** mesh (1536-atom model computed at **q** = **0**). The vertical lines show how the vibrations of *v* − SiO_2_ are sampled using the 192-atom model at **q** = **0** only (first row, corresponding to the abscissas of the scatter points in **a**)), using the 192-atom model and relying on **q** interpolation on a 3 × 3 × 3 mesh (second row), using the 1536-atom model at **q** = **0** only (third row), and using the 5184-atom model at **q** = **0** only (last row).

We start by recalling that the WTE also generalizes the AF model accounting for anharmonicity; in particular, the Lorentzian distribution (4) appearing in the WTE conductivity (1) has a FWHM determined by the anharmonic linewidths. From a computational perspective, and recalling that the size of the model determines the average energy-level spacing (6), one expects that evaluating the WTE using a finite-size model should lead to results negligibly affected by finite-size effects whenever all the anharmonic linewidths of the finite-size model are larger than its average energy-level spacing. To proceed, we first show in Fig. 3a) the frequency-linewidth distributions for the 192-, 1536-, and 5184-atom *v* − SiO_2_ models, and compare these with the average energy-level spacing. Each cloud of points represents the frequency-linewidth distribution at a given temperature for the 192-atom model, evaluated explicitly at **q** = **0** only and accounting both for third-order anharmonicity^87–91^ and for scattering due to isotopic mass disorder at natural abundance^92^ (see Methods for details). Each solid line is a coarse-grained interpolation of the frequency-linewidth distribution into a single-valued function Γ_a_[*ω*]; such a coarse graining is inspired by past work^51,93^ and validated in Fig. 11 in the Methods, where we show that evaluating the WTE conductivity using the exact frequency-linewidth distribution or linewidths determined using the coarse grained function Γ(**q**)_*s*_ = Γ_a_[*ω*(**q**)_*s*_] yields practically indistinguishable results. Clearly, in the 192-atom model a significant portion of the vibrational modes have linewidths smaller than the average energy-level spacing at room temperature and below (this happens both when vibrations are sampled at **q** = **0** only, as well as when they are more densely sampled on a 3 × 3 × 3 **q** mesh). Increasing the size of the model yields a reduction of the average energy-level spacing, and in the largest 5184-atom model most of the linewidths are larger than the average energy-level spacing already at 50 K.

To better understand the combined effect of anharmonicity and of the finite size of the model on conductivity, we recall once again that when the WTE conductivity (1) and (4) is evaluated repeating periodically a finite-size reference cell, the conductivity can be decomposed in the sum of a populations and a coherences contribution (*κ*_P_ and *κ*_C_, respectively, see Sec. *Wigner formulation of thermal transport*). We stress that the populations (coherences) conductivity can be rationalized in terms of a particle-like (wave-like) conduction mechanisms exclusively in actual crystals, where the periodicity and symmetries of the atomic structure imply that the BZ and the vibrational spectrum can be defined unambiguously^68^; consequently, the group velocities with which the particle-like phonon wavepackets propagate, and the interband spacings that characterize the wave-like tunneling of phonons, are well defined. In finite-size glass models, instead, the decomposition in populations and coherences conductivities is useful to understand finite-size effect (more on this later), but cannot be further interpreted in terms of particle-like and wave-like conduction mechanisms. In fact, such an interpretation is well defined only in crystals where the BZ and vibrational spectrum can be unambiguously defined, while in finite-size models of glasses the BZ volume decreases as the size of the model increases. Finally, we also note that the distinction between populations and coherences conductivities in finite-size models of glasses is different from the distinction between ‘propagons’ and ‘diffusons’ conductivities discussed by Allen et al.^10^ for an ideal (infinitely large) glass. The propagons and diffusons conductivities differentiate the contributions to heat transport originating from vibrational modes that propagate ballistically (akin to phonon wavepackets) from those that do not in an ideal glass; future work will aim at decomposing the total Wigner conductivity into those contributions.

In finite-size models of glasses the populations conductivity *κ*_P_ is given by the terms diagonal in the mode index ($s={s}^{{\prime} }$ in the sum appearing in Eq. (1)), since perfectly degenerate vibrational modes are absent (or a negligible fraction of the total number of modes, this because degeneracies are due to point-group symmetries^80^ and these are supposed to be absent in a realistic finite-size model of a glass). For an ideal, infinitely large model of a glass, which can be described accurately by a calculation at **q** = **0** only, one would obtain *κ*_P_ = 0, since at **q** = **0** acoustic vibrations have zero specific heat and the time-reversal symmetry of the dynamical matrix^80^ implies that the group velocities for optical vibrations are zero; the only non-zero contribution to the total conductivity would be the coherences conductivity *κ*_C_, provided some non-zero off-diagonal velocity-operator elements exists (a condition that is verified when vibrations are not Anderson-localized^9,10^).

**Fig. 4:**
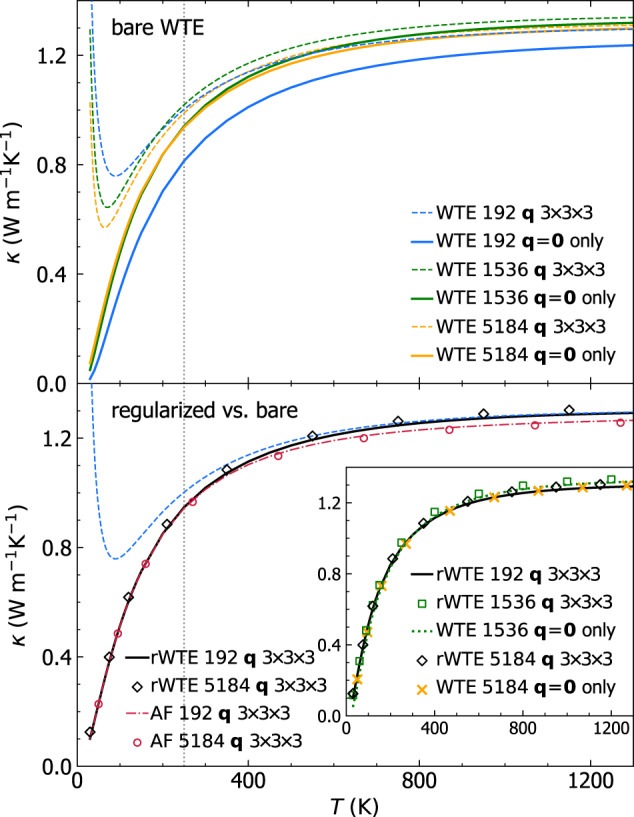
Convergence of the anharmonic Wigner conductivity in finite-size *v*-SiO_2_ models. Top, bare WTE conductivity (Eqs. (1), (4)) from the 192-atom (blue), 1536-atom (green), or 5184-atom (orange) model, and evaluated at **q** = **0** (solid lines) or on a 3 × 3 × 3 **q** mesh to extrapolate to the bulk limit (dashed lines). The crystal-like divergence of the WTE conductivity computed using the **q** mesh is a finite-size effect, occurring at lower temperatures for larger models. For *T* > 250 K, in the 1536-atom and 5184-atom models the conductivities computed at **q** = **0** are indistinguishable from those computed using the **q** mesh; for the 192-atom model, instead, employing the **q** interpolation is crucial to obtain the correct bulk limit. Bottom, regularized WTE conductivity (rWTE) for the 192- (solid black) or 5184-atom (empty diamonds) model, both computed using a 3 × 3 × 3 **q** mesh. The rWTE smoothly connects the correct low- and high-temperature limits, i.e., the fully **q**-sampled AF conductivity (as in Fig. 1, the dashed-dotted red line is the 192-atom model, empty red circles are the 5184-atom model) and the bare WTE (dashed blue), respectively. Inset: the bare WTE conductivity computed at **q** = **0** for the 1536- and 5184-atom models are practically equal to the **q**-sampled rWTE conductivity of the 192-atom model; for the 1536- and 5184-atom models the bare WTE conductivity at **q** = **0** is practically equivalent to the rWTE conductivity computed on a 3 × 3 × 3 **q** mesh.

As discussed in the previous section, a technique to extrapolate the bulk limit from finite-size models consists in relying on Fourier interpolation to sample vibrations in a *n* × *n* × *n* Born von-Karman supercell of the finite periodic reference cell, while remaining aware of the limitations stemming from having a disorder length scale limited by the size of the reference cell. Figure 3b shows that such Fourier interpolation allows to greatly improve the accuracy of the thermodynamic predictions for the 192-atom model; specifically, the vibrational density of states (vDOS) of the 192-atom model computed using a 3 × 3 × 3 **q** mesh is in very good agreement with the vDOS of both the 1536- and 5184-atom models computed at **q** = **0** only. For reference cells larger than a certain size, one expects the conductivities computed at **q** = **0** only or using a *n* × *n* × *n* Fourier interpolation to be practically indistinguishable. To support this expectation, we note that the sum over the modes appearing in Eq. (1) contains ${(3{N}_{{{{\rm{at}}}}})}^{2}$ term, for a disordered system without degeneracies 3*N*_at_ diagonal ($s={s}^{{\prime} }$) terms are ‘populations’ that vanish at **q** = **0** due to time-reversal symmetry, the remaining ${(3{N}_{{{{\rm{at}}}}})}^{2}-3{N}_{{{{\rm{at}}}}}$ terms are ‘coherences’. The difference between a conductivity calculation at **q** = **0** only and one on the *n* × *n* × *n* mesh is expected to go to zero with a speed directly correlated with the ratio between the number of diagonal elements and total number of elements, i.e., $\frac{3{N}_{{{{\rm{at}}}}}}{{(3{N}_{{{{\rm{at}}}}})}^{2}}=\frac{1}{3{N}_{{{{\rm{at}}}}}}$. In order to test the accuracy of the bulk-limit extrapolations performed using the **q** interpolation, and to verify the aforementioned expectations, one has to perform calculations in models having different sizes (ideally in one large model that already describes the bulk limit and for which the **q** interpolation is therefore not needed, and in one small model where using or not the interpolation technique is expected to yield appreciable effects). In Fig. 4 (upper panel) we compare the ‘bare’ WTE conductivity (Eq. (1) and (4)) obtained using the 192-atom model (blue), the 1536-atom model (green), or the 5184-atom model (orange). For each model two conductivity calculations are performed: sampling **q** = **0** only (solid lines) or sampling a 3 × 3 × 3 **q** mesh (dashed lines); the most dramatic difference between these two calculations occurs in the low-temperature limit, where they yield conductivities having opposite trends. The divergence of the WTE conductivity computed using the Fourier interpolation is a finite-size effect arising from the periodic-boundary conditions (a reminiscence of the divergence at low temperatures of the conductivity of bulk crystals that is cutoff in real crystals by the scattering with grains’ or samples’ boundaries); such an effect occurs at lower temperatures for larger modes; thus, it is expected to vanish in the ideal glass limit, i.e., for *N*_at_ → *∞* *κ*(*T*) in the above-the-plateau regime is expected to follow the same trend observed in the calculation at **q** = **0** and in experiments (more on this later). We note, in passing, that this reasoning also explains the opposite trend of the broadening versus AF conductivity curve computed at **q** = **0** only or using the Fourier interpolation shown in Fig. 2. In fact, in the limit of vanishing broadening *η* → 0 only the term $s={s}^{{\prime} }$ determines the value of the sum in Eq. (1), yielding $\scriptstyle \kappa \approx \frac{1}{{{{\mathcal{V}}}}{N}_{C}}{\sum }_{{{{\bf{q}}}}s}C[\omega {({{{\bf{q}}}})}_{s}]\frac{| | {\mathsf{v}}{({{{\bf{q}}}})}_{s,s}| {| }^{2}}{\eta }$. Such a limiting expression for the conductivity diverges when the Fourier interpolation is adopted, since at **q** ≠ **0** the diagonal velocity-operator elements are non-zero; in contrast, in a calculation at **q** = **0** only the time-reversal symmetry implies that the diagonal velocity-operator elements are zero; thus, in this case the conductivity approaches zero when the broadening goes to zero.

We note that for the 1536- and 5184-atom models at temperatures higher than ~ 100 K the WTE conductivity obtained relying on the Fourier interpolation is practically equal to the conductivity obtained evaluating the WTE conductivity at **q** = **0** only. In contrast, for the 192-atom model the conductivity obtained from a calculation at **q** = **0** only is always significantly different from that obtained using a computationally converged 3 × 3 × 3 **q** mesh (we have verified that using a denser 5 × 5 × 5 **q** mesh yields results practically equivalent to the 3 × 3 × 3 **q** mesh). Most importantly, for temperatures higher than 250 K the WTE conductivity obtained from the 192-atom model using the Fourier interpolation is practically indistinguishable from the conductivities obtained from the 1536- and 5184-atom models, confirming that the interpolation allows to accelerate convergence in the calculation of the bulk limit.

The considerations above show that in finite-size models the WTE conductivity with Fourier interpolation is accurate up to a lower-bound temperature *T*_*L*_ (roughly defined as the temperature at which the temperature-conductivity curve changes concavity). Below *T*_*L*_ finite-size effects lead to a crystal-like divergence, which emerges as a consequence of having a significant number of vibrational modes with anharmonic linewidths ℏΓ(**q**)_*s*_ smaller than ℏΔ*ω*_avg_ (see Fig. 3); from a microscopic viewpoint, this implies that couplings in the distribution (4) between quasi-degenerate eigenstates—which are present in an ideal glass, see Sec. *Protocol to evaluate the harmonic Allen-Feldman conductivity with finite-size models*—are not correctly accounted for. Thus, the finite-size model fails to represent the harmonic conduction described by the distribution (5) and accurate for an ideal glass at low temperature (here ‘low temperature’ must be interpreted keeping into account that this work is focused on the above-the-plateau temperature regime, i.e.*, T* > 30*K*). This limitation can be overcome relying on the fact that in the low-temperature limit anharmonicity progressively phases out; thus, the AF model becomes increasingly more accurate and can be evaluated using the protocol discussed in the previous section. Therefore, we introduce a regularization protocol for the WTE that allows to determine its bulk limit using Fourier interpolation, and accounts for the prescriptions needed to correctly evaluate the low-temperature harmonic limit discussed before. Specifically, we choose for our protocol a Voigt profile^94^—a two-parameter distribution ${{{{\mathcal{F}}}}}_{[\Gamma {({{{\bf{q}}}})}_{s}+\Gamma {({{{\bf{q}}}})}_{{s}^{{\prime} }},\eta ]}$ obtained as a convolution between a Lorentzian with FWHM $\Gamma {({{{\bf{q}}}})}_{s}+\Gamma {({{{\bf{q}}}})}_{{s}^{{\prime} }}$ and a Gaussian with variance *η*^2^*π*/2 (see Methods for details)—in place of the one-parameter distribution ${{{{\mathcal{F}}}}}_{[\Gamma {({{{\bf{q}}}})}_{s}+\Gamma {({{{\bf{q}}}})}_{{s}^{{\prime} }}]}$ appearing in Eq. (1). By doing so Eq. (1) reduces to the AF harmonic limit at low temperatures (where $\Delta {\omega }_{{{{\rm{avg}}}}} \sim \eta \gg \frac{1}{2}(\Gamma {({{{\bf{q}}}})}_{s}+\Gamma {({{{\bf{q}}}})}_{{s}^{{\prime} }})$), and to the anharmonic WTE at intermediate and high temperatures (where $\Delta {\omega }_{{{{\rm{avg}}}}} \sim \eta \ll \frac{1}{2}(\Gamma {({{{\bf{q}}}})}_{s}+\Gamma {({{{\bf{q}}}})}_{{s}^{{\prime} }})$). Hereafter the conductivity computed using the Fourier interpolation (to determine the bulk limit) and the Voigt distribution (to correctly describe the low-temperature harmonic limit) will be referred to as ‘regularized WTE’ (rWTE), to distinguish it from the ‘bare’ WTE (Eq. (1) with the Lorentzian distribution (4)). As shown in the bottom panel of Fig. 4, the rWTE conductivity reduces to the AF harmonic conductivity for temperatures lower than *T*_*L*_, and to the bare anharmonic WTE conductivity for temperature higher than *T*_*L*_, smoothly connecting these two limits. The parameter *η* entering in the Voigt profile has to be chosen equal to the value determining the beginning of the convergence plateau (see vertical dotted lines in Fig. 2). Using a smaller value of *η* would not allow to recover in the harmonic limit (Γ(**q**)_*s*_ → 0 ∀ **q**, *s*) the bulk AF conductivity discussed in Sec. *Protocol to evaluate the harmonic Allen-Feldman conductivity with finite-size models*, since in this limit the Voigt profile would reduce to a Gaussian too narrow to capture the couplings between neighboring eigenstates. Conversely, choosing a larger value of *η* would artificially alter the effects of anharmonicity, since the Voigt profile reduces to the anharmonic Lorentzian distribution when $\eta \ll \frac{1}{2}(\Gamma {({{{\bf{q}}}})}_{s}+\Gamma {({{{\bf{q}}}})}_{{s}^{{\prime} }})$ and a too large value of *η* (e.g., orders of magnitude larger than Δ*ω*_avg_) would cause an unnecessary, artificial renormalization of anharmonic effects. In fact, when Δ*ω*_avg_ ≪ Γ(**q**)_*s*_ ∀ **q**, *s* the linewidths are large enough to describe couplings between different vibrational eigenstates, and thus do not need to be renormalized to enforce the harmonic AF limit (we recall that in the WTE framework at finite temperature the couplings and related wave-like tunneling events are not limited to quasi-degenerate eigenstates). In practice, using the rWTE protocol with this prescription for *η* ensures that the low-temperature harmonic AF limit is accurately described, and the effects of anharmonicity are considered only when they are not altered by finite-size effects. The inset of Fig. 4 highlights that the rWTE conductivities evaluated on a 3 × 3 × 3 **q**-mesh for the 192-, 1536-, and 5184-atom models are practically indistinguishable. We note in passing that for the 1536- and 5184-atom models the WTE conductivity evaluated at **q** = **0** only is equal to the rWTE conductivity evaluated on a 3 × 3 × 3 **q** mesh, showing that models containing more than 1500 atoms are large enough to describe the bulk limit without relying on the **q** interpolation and on the regularization; these considerations are further discussed in Fig. 12 in the Methods. In summary, we have shown that the rWTE regularization protocol greatly accelerates the convergence of the conductivity calculation, allowing to determine the conductivity of *v* − SiO_2_ at non-cryogenic temperatures using models containing less than 200 atoms and having linear size of about 1.5 nanometre. Our findings are supported by past work: refs. ^63,95^ showed that models of vitreous silica containing 72 or 144 atoms can be used to rationalize experimental measurements of vibrational properties relevant for heat conduction at non-cryogenic temperatures; the experiments of refs. ^96,97^ showed that the typical propagation lengthscale of vibrations in silica are in the sub-nanometre region at non-cryogenic temperatures. More generally, our approach is expected to apply equally well to other ‘strongly disordered’ amorphous solids, in which structural disorder is significant already at the sub-nanometre length scale (e.g., amorphous Al_2_O_3_, as discussed in ref. ^98^). It is also worth mentioning that amorphous solids with weaker or more complex disorder, e.g., involving phase separation^99,100^, or structural motifs with a characteristic lengthscale of few nanometres^101^, are expected to require primitive cells including these motifs and thus containing thousands of atoms.

### First-principles calculations and comparison with experiments

Hitherto, no theoretical work has managed to evaluate the thermal conductivity of *v*-SiO_2_ (and more generally of any amorphous solid) from first principles and accounting for the interplay between anharmonicity, disorder, and the Bose-Einstein statistics of atomic vibrations. More precisely, past works studied the thermal conductivity of *v*-SiO_2_ using a variety of approaches, including: (i) the AF model in combination with empirical^102^ or semi-empirical^21^ potentials; (ii) classical molecular dynamics^21,24,35,36^; (iii) first-principles molecular dynamics^26^. It is worth highlighting that the determination of the thermal conductivity of amorphous solids from first-principles molecular dynamics is a recent advance in the field^26,32,33^. These pioneering works focused on the high-temperature regime, where the difference between the actual quantum Bose-Einstein occupation numbers of vibrations and the classical (equipartition-determined) occupation numbers implicit in the molecular-dynamics simulations^39^ is minimal. We have seen the aforementioned rWTE protocol allows to accurately determine the bulk thermal conductivity of glasses also at temperatures where quantum effects are relevant and using models with a size ( ≲ 200 atoms) that is within the reach of first-principles techniques; so we now employ these to compute the conductivity of *v*-SiO_2_.

**Fig. 5:**
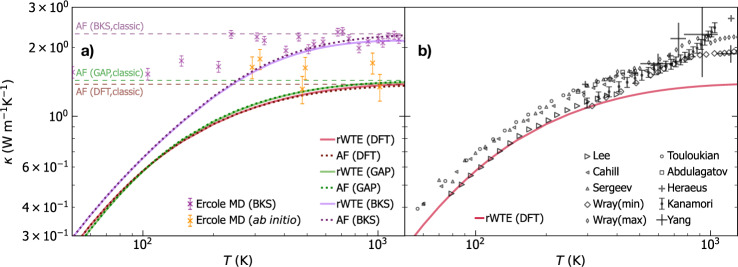
Thermal conductivity of *v*-SiO_2_: theory vs experiments. **a** Solid (dotted) lines are the rWTE (AF) conductivities for a 192-atom (`192(D)') model of *v*-SiO_2_ generated using first-principles techniques^65^, repeated periodically 3 × 3 × 3 times and studied using first-principles DFT calculations (red), a GAP potential (green), or the BKS potential (purple). The dashed lines are the classical AF conductivities (same color code), corresponding to vibrational modes having specific heat always equal to *k*_*B*_^39^. Cyan and purple scatter points are predictions from first-principles and classical (BKS-based) molecular dynamics simulations, respectively, from Ercole et al.^26^. These MD calculations are governed by classical equipartition^39^ and generally agree with the corresponding classical AF conductivities (the increasingly larger differences between MD and classical AF observed lowering temperature may be due to the decrease of the accuracy with which atomic vibrations are sampled in MD simulations when temperature is lowered). Importantly, in the high-temperature limit, when the correct quantum Bose-Einstein statistics of vibrations yields a specific heat approaching the classical limit, the MD calculations are in good agreement with the corresponding (first-principles- or BKS-based) rWTE predictions. **b** Scatter points are experiments from Wray et al.^143^, Kanamori et al.^107^, Touloukian et al.^144^, Sergeev et al.^145^, Cahill^146^, Lee and Cahill (190 nm sample)^147^, Abdulagatov et al.^148^, Yang et al.^106^, and Heraeus^139^. The red line is the (most accurate) first-principles rWTE conductivity, which is in agreement with experiments at temperatures where radiative effects are negligible (*T* ≲ 450 K).

We show in Fig. 5a the bulk rWTE conductivity (solid lines) of a ‘192(D)’ model of *v*-SiO_2_, which contains 192 atoms and was generated relying on density-functional theory (DFT)^62,65^ (this model is different from the 192-atom model generated with GAP and discussed in the previous sections; to avoid confusion we will henceforth denote the 192-atom model discussed in the previous sections with ‘192(G)’). To assess the effects of anharmonicity on the conductivity, also the harmonic AF conductivity is shown (dotted lines). All the parameters entering in the rWTE or AF expressions have been evaluated either from first-principles (red, see Methods for details), or using a state-of-the-art GAP potential^60^ (green), or using the well known semi-empirical BKS potential^66,67^ (purple). In all these cases we used a broadening *η* = 4 cm^−1^ for the Voigt distribution, this value was determined from a convergence test analogous to that reported in the upper-left panel of Fig. 2. Orange and purple scatter points are results from first-principles GKMD (using the PBE functional) and classical GKMD using the BKS potential, respectively, by Ercole et al.^26^; these are meant to be accurate at high temperature where the quantum Bose-Einstein occupations approach the classical (equipartition) occupations underlying the GKMD simulations^39^. Importantly, Fig. 5a shows that the conductivity of *v*-SiO_2_ is negligibly affected by anharmonicity. In fact, the rWTE perturbatively accounts for anharmonicity at the lowest cubic order, and yields a thermal conductivity that is (i) practically indistinguishable from that obtained employing the harmonic Allen-Feldman theory over the entire temperature range analyzed (30–1300 K); (ii) in good agreement at high temperature (*T* ≳ 500 K) with the conductivity obtained by Ercole and Baroni using GKMD^26^, which accounts for anharmonicity exactly (in the high-temperature limit our rWTE calculations, either from first-principles or based on the BKS potential, are in very good agreement with the corresponding GKMD predictions. We also note that the first-principles GKMD results of ref. ^26^ were obtained from a 52-ps-long simulation and using a 432-atom model, while the computationally cheaper BKS-based GKMD calculations of ref. ^26^ were obtained from a 1 ns-long calculation and using 432-atom and 10800-atom models (compatible results were obtained for these different sizes). Clearly, shorter first-principles GKMD calculations have a larger uncertainty; we highlight how for T>500 K Ercole’s first-principles GKMD calculations oscillate above and below the classical limit of our AF (DFT) calculations. Figure 5a reinforces the notion that the quantum Bose-Einstein statistics of vibrations plays a crucial role in determining the thermal conductivity^39^ at low temperatures, since the conductivities computed accounting for the quantum statistics (and for the correct bulk limit) increase up to saturation with temperature, while the GKMD conductivities are governed by classical equipartition and are roughly independent from temperature. In addition, we highlight how studying the same 192(D) structure using first-principles techniques (red) or the GAP potential (green) yields very similar results, further endorsing the notion that machine-learned GAP potentials can have an accuracy comparable to that of first-principles techniques^103,104^. In contrast, using the semi-empirical BKS potential yields results that are significantly different from those obtained from first-principles. Importantly, Fig. 5b shows that the first-principles rWTE conductivity is in good agreement with experiments in the temperature range from 50 to ~450 K; at higher temperatures the rWTE predicts a saturating trend for the conductivity (which mirrors the saturating trend of the specific heat, see Fig. 13 in the Methods), while experiments display an increasing drift. Past experimental work (e.g., ref. ^105^ and references therein) hinted that radiative energy transfer causes an increase of the thermal conductivity in vitreous silica at high temperature, and highlighted how accurately distinguishing the conduction and radiation contributions to heat transfer is particularly challenging^106^ (among the various sources of difficulty, refs. ^107,108^ mentioned how the interplay between conduction and radiation depends from the size and shape of the sample used in the experiment). Thus, in Fig. 5 the discrepancy between theory and experiments at high temperature might be due to having non-negligible radiative contributions in the experiments^106,107^, and to not accounting for these radiative contributions in our calculations.

**Fig. 6:**
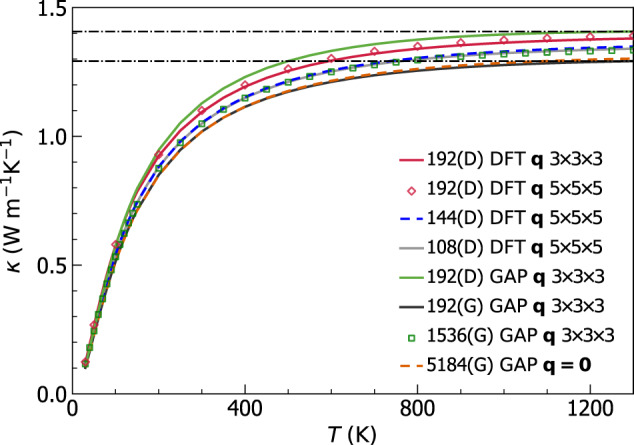
rWTE conductivity of various *v*-SiO_2_ models. Solid red is the first-principles rWTE conductivity of the 192-atom model generated relying on first-principles calculations^65^, computed using a 3 × 3 × 3 **q** mesh; using a denser 5 × 5 × 5 **q** mesh does not yield significant changes. The solid-gray and dashed-blue lines show the first-principles rWTE conductivity computed using a 5 × 5 × 5 **q** mesh for a 108-^62^ and 144-atom^63^ model, respectively; both models have been generated relying on first-principles calculations. The conductivities of the 192- (black line), 1536- (empty green squares), and 5184-atom (dashed orange) models discussed in Fig. 4 are also reported (these are labeled with the suffix `(G)' to distinguish them from the structures generated relying on first-principles techniques, which are labeled with `(D)'); the horizontal lines highlight the 9% difference between the rWTE conductivities of the 192(D) and 192(G) models, both described with GAP.

In Fig. 5, the bulk limit has been computed using a 3 × 3 × 3 Fourier-interpolation mesh (thus corresponding to vibrations in a system containing 192 ⋅ 3^3^ = 5184 atoms); Fig. 6 demonstrates that using this sampling achieves computational convergence, since increasing the mesh to 5 × 5 × 5 yields practically indistinguishable results (empty red diamonds refer to a 5 × 5 × 5 mesh, solid red line refers to a 3 × 3 × 3 mesh). Figure 6 also shows that studying both the 192(D) and the 192(G) models with GAP gives high-temperature limits for their rWTE conductivities differing by about 9%. Such a small difference is particularly reassuring, given the two different methods employed to produce these models (bond switching in the first case^62,65^ and melt-quench in the second case^60^). Moreover, Fig. 6 shows that the rWTE conductivity computed from first principles using the 192(D) model (solid red) is very similar to the conductivity computed from first-principles density-functional theory using models containing 108^62^ (solid gray) or 144 atoms^63,64^ (dashed blue), both generated relying on first-principles calculations (therefore, we henceforth refer to these models to as ‘108(D)’ and ‘144(D)’, respectively).

### Velocity operator, anharmonicity, trend of *κ*(*T*)

**Fig. 7:**
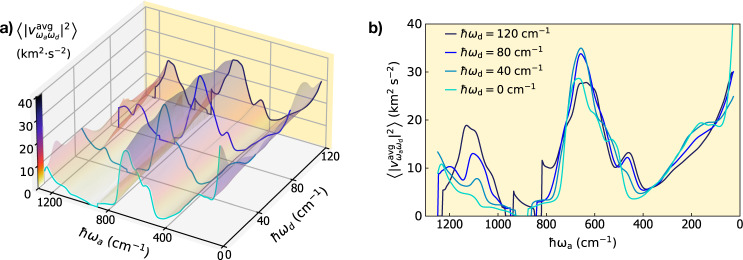
Velocity operator of vitreous silica and conductivity saturation with temperature. **a** Average square modulus of the velocity-operator elements $\langle | {{\mathsf{v}}}_{{\omega }_{{{{\rm{a}}}}}{\omega }_{{{{\rm{d}}}}}}^{{{{\rm{avg}}}}}{| }^{2}\rangle$ for the 192(D) model of *v*-Si*O*_2_, computed from first principles and represented as a function of the energy differences ($\hslash {\omega }_{{{{\rm{d}}}}}=\hslash (\omega {({{{\bf{q}}}})}_{s}-\omega {({{{\bf{q}}}})}_{{s}^{{\prime} }})$) and averages ($\hslash {\omega }_{{{{\rm{a}}}}}=\hslash \frac{\omega {({{{\bf{q}}}})}_{s}+\omega {({{{\bf{q}}}})}_{{s}^{{\prime} }}}{2}$) of the two coupled eigenstates (having wavevector **q** and modes $s,{s}^{{\prime} }$; see text for details). The one-dimensional projections in (**b**) show that the elements $\langle | {{\mathsf{v}}}_{{\omega }_{{{{\rm{a}}}}}{\omega }_{{{{\rm{d}}}}}}^{{{{\rm{avg}}}}}{| }^{2}\rangle$ are almost unchanged at a given average frequency for increasingly large energy differences. For increasingly larger temperatures, these almost-constant elements drive the saturation the rWTE conductivity (Eq. (1) with the Voigt distribution), yielding results very close to the Allen-Feldman conductivity curve (Fig. 5), which is determined exclusively by velocity-operator elements with ℏ*ω*_d_ → 0.

Now we want to rely on the results obtained for *v*-SiO_2_ to gain general insights on how anharmonicity affects the high-temperature trend for the *κ*(*T*) curve in glasses. We start by recalling that, according to the expression for the rWTE conductivity (Eq. (1) with the Voigt distribution discussed in Sec. *Extension of the protocol to evaluate the anharmonic Wigner conductivity*), heat conduction in strongly disordered glasses is mainly determined by couplings between vibrational eigenstates. At low temperature anharmonicity phases out and the rWTE conductivity reduces to the AF limit, where only couplings between quasi-degenerate vibrational eigenstates contribute to heat transport (we recall that representing the Dirac delta appearing in the AF expression with a light-tailed Gaussian ensures that this analytical property is satisfied in numerical calculations). The strengths of these AF couplings is determined exclusively by the square modulus of the corresponding quasi-degenerate velocity-operator elements ($\frac{1}{3}\parallel {\mathbf{v}}{({{{\bf{q}}}})}_{s,{s}^{{\prime} }}{\parallel }^{2}$ with $| \omega {({{{\bf{q}}}})}_{s}-\omega {({{{\bf{q}}}})}_{s}^{{\prime} }| \lesssim \Delta {\omega }_{{{{\rm{avg}}}}}$), and consequently all the temperature dependence of the AF conductivity is inherited by the quantum specific heat (in this work the frequencies and velocity operators are considered independent from temperature, since we employ the standard approximation of considering the force constants to be independent from temperature, details on the accuracy of this approximation in vitreous silica are discussed in the Methods). In contrast, when the linewidths are larger than the average energy-level spacing (e.g., at high temperature, see Fig. 3) the rWTE conductivity is determined by velocity-operator elements coupling eigenstates having energy difference spanning the entire energy range. More precisely, as temperature and anharmonic linewidths increase, the Voigt profile reduces to a fat-tailed Lorentzian distribution (4) with FWHM determined by the linewidths. Such a fat-tailed distribution becomes broader as temperature increases (Fig. 3); when determining the conductivity, this gives more weight to velocity-operator elements with increasingly larger frequency difference. Therefore, at high temperature (when the specific heat reduces to the constant classical limit *k*_*B*_), the variation of the elements of the velocity operator with respect to the energy differences $\hslash {\omega }_{{{{\rm{d}}}}}=\hslash (\omega {({{{\bf{q}}}})}_{s}-\omega {({{{\bf{q}}}})}_{{s}^{{\prime} }})$ determines the scaling of the rWTE conductivity: matrix elements increasing or decreasing with *ω*_*d*_ imply a conductivity increasing or decreasing with temperature. From this reasoning and from the saturating trend of the temperature-conductivity curve for vitreous silica shown in Fig. 5, we expect the velocity-operator elements for *v*-SiO_2_ to be approximately constant with respect to *ω*_*d*_. To verify this prediction, we plot in Fig. 7a the velocity-operator elements for *v*-SiO_2_ (we show the velocity operator for the 192(D) model and computed from first principles; the other models yield practically indistinguishable results when analyzed from first principles or using GAP) as a function of the energy difference ℏ*ω*_d_ and of the energy average $\hslash {\omega }_{{{{\rm{a}}}}}=\hslash (\omega {({{{\bf{q}}}})}_{s}+\omega {({{{\bf{q}}}})}_{{s}^{{\prime} }})/2$:

7 $$\begin{array}{rcl}\left\langle | {{\mathsf{v}}}_{{\omega }_{{{{\mathrm{a}}}}}{\omega }_{{{{\mathrm{d}}}}}}^{{{{\mathrm{avg}}}}}{| }^{2}\right\rangle &=&{[{{{\mathcal{G}}}}({\omega }_{{{{\rm{a}}}}},{\omega}_{{{{\mathrm{d}}}}})]}^{-1}\frac{1}{{{{\mathcal{V}}}}{N}_{{{{\mathrm{c}}}}}}\mathop{\sum}\limits_{{{{\mathbf{q}}}},s,{s}^{{\prime} }}\frac{\parallel {{\mathbf {v}}}{({{{\mathbf{q}}}})}_{s,{s}^{{\prime} }}{\parallel }^{2}}{3}\\ &\times &\delta \left({\omega}_{{{{\rm{d}}}}}-(\omega {({{{\bf{q}}}})}_{s}-\omega {({{{\mathbf{q}}}})}_{{s}^{{\prime} }})\right)\delta \left({\omega}_{{{{\mathrm{a}}}}}-\frac{\omega {({{{\bf{q}}}})}_{s}+\omega {({{{\mathbf{q}}}})}_{{s}^{{\prime}}}}{2}\right);\end{array}$$

${{{\mathcal{G}}}}({\omega }_{{{{\rm{a}}}}},{\omega }_{{{{\rm{d}}}}})$ is a density of states that serves as normalization (see Methods for details). The one-dimensional projections in Fig. 7b show that these velocity-operator elements are almost constant at varying ℏ*ω*_d_ for all values of ℏ*ω*_a_. These findings validate the reasoning above: the variation of the velocity-operator elements $\frac{1}{3}\parallel {{\mathbf{v}}}{({{{\bf{q}}}})}_{s,{s}^{{\prime} }}{\parallel }^{2}$ with respect to the energy differences ℏ*ω*_d_ determines how the conductivity varies with increasing temperature, implying that a saturating temperature-conductivity curve is obtained when most of the velocity-operator elements do not vary with ℏω_d_. The reasoning above also explains the small or negligible effects of anharmonicity on the conductivity of *v*-SiO_2_ discussed before, since having a velocity operator displaying a negligible dependence on ℏ*ω*_d_ implies that the rWTE conductivity does not vary appreciably when the linewidths (broadening of the Lorentzian distribution) vary, and therefore it does not significantly differ from the AF conductivity (computed using a constant broadening *η* and determined only by velocity-operator elements in the limit ℏ*ω*_d_ → 0, see Methods for details).

### Thermal diffusivity

In order to gain further insight on the microscopic mechanisms underlying conduction, it is useful to resolve how each vibrational mode contributes to transport; i.e., the quantity of heat that it carries and the rate at which it diffuses. It is possible to extract the contribution of a single vibration to thermal transport by factorizing the single-vibration specific heat *C*(**q**)_*s*_ in the regularized rWTE conductivity (1) recasting it as $\kappa =\frac{1}{{{{\mathcal{V}}}}{N}_{{{{\rm{c}}}}}}{\sum }_{{{{\bf{q}}}}s}C{({{{\bf{q}}}})}_{s}D{({{{\bf{q}}}})}_{s}$, with *D*(**q**)_*s*_ being the ‘anharmonic thermal diffusivity’. The expression for *D*(**q**)_*s*_ is determined by such factorization and by the requirement that in the coherences’ coupling between two vibrations (**q**)_*s*_ and ${({{{\bf{q}}}})}_{{s}^{{\prime} }}$ each contributes to the coupling with a weight equal to the relative specific heat^41^ (e.g., for vibration (**q**)_*s*_ the weight is $\frac{C{({{{\bf{q}}}})}_{s}}{C{({{{\bf{q}}}})}_{s}+C{({{{\bf{q}}}})}_{{s}^{{\prime} }}}$, and correspondingly for vibration ${({{{\bf{q}}}})}_{{s}^{{\prime} }}$ the weight is $\frac{C{({{{\bf{q}}}})}_{{s}^{{\prime} }}}{C{({{{\bf{q}}}})}_{s}+C{({{{\bf{q}}}})}_{{s}^{{\prime} }}}$):

8 $$\begin{array}{lll}D{({{{\bf{q}}}})}_{s}\,=\,\mathop{\sum}\limits_{{s}^{{\prime} }}\frac{\omega {({{{\bf{q}}}})}_{s}+\omega {({{{\bf{q}}}})}_{{s}^{{\prime} }}}{2[C{({{{\bf{q}}}})}_{s}+C{({{{\bf{q}}}})}_{{s}^{{\prime} }}]}\left[\frac{C{({{{\bf{q}}}})}_{s}}{\omega {({{{\bf{q}}}})}_{s}}+\frac{C{({{{\bf{q}}}})}_{{s}^{{\prime} }}}{\omega {({{{\bf{q}}}})}_{{s}^{{\prime} }}}\right]\frac{\parallel {{\mathbf{v}}}{({{{\bf{q}}}})}_{s,{s}^{{\prime} }}{\parallel }^{2}}{3}\\ \qquad\qquad\times \,\pi {{{{\mathcal{F}}}}}_{[\Gamma {({{{\bf{q}}}})}_{s}+\Gamma {({{{\bf{q}}}})}_{{s}^{{\prime} }},\eta ]}(\omega {({{{\bf{q}}}})}_{s}-\omega {({{{\bf{q}}}})}_{{s}^{{\prime} }}).\end{array}$$

**Fig. 8:**
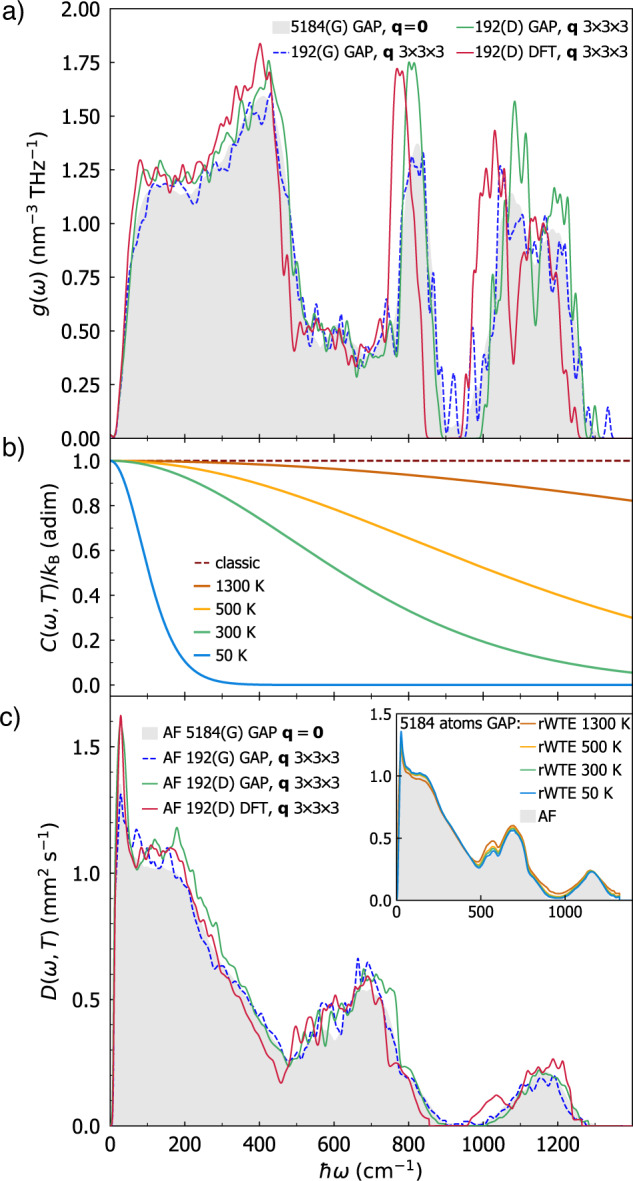
Vibrational DOS, specific heat, and diffusivity of vitreous silica. **a** Red and green are the vDOS for the 192(D)^65^ model (192-atom model generated from first-principles DFT simulations), obtained evaluating the frequencies of such model either from first principles or from the GAP potential, and using a 3 × 3 × 3 **q** mesh. The dashed blue line and gray area are the vDOS of the GAP-generated^60^ 192- and 5184-atom models, computed using GAP as in Fig. 4 (i.e., relying on a 3 × 3 × 3 **q** mesh in the former case, and at **q** = **0** only in the latter case). **b** Solid lines are the quantum harmonic specific heat as a function of frequency and temperature from Eq. (2). The dashed line are the classical specific heat. **c** Harmonic Allen-Feldman thermal diffusivity computed from Eq. (8) using Γ(**q**)_*s*_ = 0 ∀ **q**, *s* and with the models discusses in **a** (same parameters and color code); the inset shows that in *v*-SiO_2_ (5184(G) model) the AF diffusivity is very similar to the anharmonic diffusivity (full Eq. (8)).

The goal of this decomposition is to resolve the rate at which the heat carried by a vibration with wavevector **q** and mode *s* diffuses. We note that Eq. (8) accounts for the effects of anharmonicity on vibrations’ diffusion by means of the linewidths, depends on temperature through both the specific heat and the linewidths, and applies to both glasses and crystals—in the former case the wavevector **q** is just a label without direct physical meaning (we will discuss later that for glasses the diffusivity has to be represented as a function of frequency to be well defined), while in the latter case such an expression is accurate if and only if the SMA is accurate and *η* = 0 is used (this last condition implies that the Voigt distribution analytically reduces to the Lorentzian distribution (4)). It is worth mentioning that in the case of simple crystals, characterized by *κ*_*P*_ ≫ *κ*_*C*_, the term ${s}^{{\prime} }=s$ in Eq. (8) yields the well known expression obtained from Peierls’s theory, which interprets the diffusivity (averaged over the three Cartesian directions) as $D{({{{\bf{q}}}})}_{s}=\frac{1}{3}| | {{\mathbf{v}}}{({{{\bf{q}}}})}_{s,s}| {| }^{2}\tau {({{{\bf{q}}}})}_{s}$, where **v**(**q**)_*s*,*s*_ is the free propagation velocity of the particle-like heat carrier with wavevector **q** and mode *s*, and *τ*(**q**)_*s*_ is the inter-collision time. In finite-size models of glasses it is most informative to represent the diffusivity as a function of frequency, first because the specific heat of a vibration depends on its frequency *ω* (*C*(**q**)_*s*_ = *C*[*ω*(**q**)_*s*_], see Eq. (2)), and second because the vibrational frequencies determine measurable quantities such as the vibrational density of states (in contrast, as mentioned before, in finite-size models of glasses quantities such as the wavevectors **q** span a BZ that depends on the model and are used only as a mathematical tool in the determination of the bulk limit). Thus, we represent the thermal diffusivity as a function of frequency with $D(\omega ,T)={[g(\omega ){{{\mathcal{V}}}}{N}_{{{{\rm{c}}}}}]}^{-1}{\sum }_{{{{\bf{q}}}},s}D{({{{\bf{q}}}})}_{s}\delta (\omega -\omega {({{{\bf{q}}}})}_{s})$ (here $g(\omega )={({{{\mathcal{V}}}}{N}_{{{{\rm{c}}}}})}^{-1}{\sum }_{{{{\bf{q}}}},s}\delta (\omega -\omega {({{{\bf{q}}}})}_{s})$ is the vibrational density of states (vDOS), which can be considered independent from temperature, as also shown in Fig. 13 in the Methods; the Dirac *δ* is broadened with a Gaussian distribution having a broadening determined from the convergence test discussed in Sec. *Protocol to evaluate the harmonic Allen-Feldman conductivity with finite-size models*). In the low-temperature and infinite-reference-cell limit, Eq. (8) reduces to the temperature-independent harmonic diffusivity introduced by Allen and Feldman^8^. This follows from the properties of the Voigt distribution ${{{{\mathcal{F}}}}}_{[\Gamma {({{{\bf{q}}}})}_{s}+\Gamma {({{{\bf{q}}}})}_{{s}^{{\prime} }},\eta ]}$ discussed in Sec. *Extension of the protocol to evaluate the anharmonic Wigner conductivity*; in the following the dependence from temperature will be shown explicitly for the sake of clarity. In this frequency-dependent representation the conductivity reads

9 $$\kappa (T)=\int\nolimits_{0}^{\infty }g(\omega )C(\omega ,T)D(\omega ,T)d\omega ,$$

and intuitively allows to resolve the contribution of vibrations with frequency *ω* to heat transport through their density of states *g*(*ω*), the heat carried *C*(*ω*, *T*), and the diffusion rate *D*(*ω*, *T*). We report in Fig. 8 all these quantities. In panel a) we show the vDOS for the 192(D) *v*-SiO_2_ model computed using first principles calculations (red) or the GAP potential (green). The main difference between these calculations is that the GAP potential slightly stretches the high-frequency part of the spectrum toward higher values; the effects on the thermal conductivity of such a slight stretch of vibrational energies is barely appreciable, as shown in Fig. 5. The dashed-blue line and the gray area are the vDOS for the 192(G) and 5184(G) models, respectively; both these vDOS are obtained using GAP, the former is computed using a 3 × 3 × 3 **q** interpolation mesh, while the latter is computed at **q** = **0** only. The similarity between these two curves further supports the usage of the **q** interpolation technique to sample more accurately the vibrations in a glass. Figure 8a shows that the vDOS is slightly affected by the method used to generated the *v*-SiO_2_ model, with the 192(G) and 5184(G) models generated using the melt-quench method^60^ leading to vDOS slightly different from the 192(D) model, which was generated using the bond-switching technique^62,65^. In panel b of Fig. 8 we report the quantum harmonic specific heat as a function of frequency, showing how increasing temperature populates vibrational modes of increasingly larger frequency that consequently contribute to transport. We highlight how, for all the temperatures considered, the quantum specific heat differs from the constant classical (equipartition) limit (obtained letting *T* → *∞* in Eq. (2)); this reiterates the important role of quantum statistics of vibrations in thermal transport^39^. Panel c shows the harmonic AF diffusivity (Eq. (8) using Γ(**q**)_*s*_ = 0 ∀ **q**, *s*) for the models of *v*-SiO_2_ discussed in panel a) (same parameters and color code are used). Considerations analogous to those for the vDOS in panel a) hold: the method used to generated the *v*-SiO_2_ model has a small but appreciable effect on the diffusivity, with the 192(D) model generated using the bond-switching method having a larger diffusivity at low frequency. Overall, the combined variations of vDOS and diffusivity due to the technique used to generate the model, or the approach (first-principles calculations or GAP) used to evaluate frequencies and velocity operators, yield differences on the thermal conductivity within 9%, as shown before in Fig. 6. The inset of panel c) shows that the anharmonic diffusivity (colored lines, computed from Eq. (8)) changes very little with temperature, and is practically very similar to the temperature-independent AF diffusivity (gray area, obtained using Eq. (8) with Γ(**q**)_*s*_ = 0 ∀ **q**, *s* and *η* determined from the convergence test detailed in Fig. 2); the inset shows calculations with GAP on the 5184(G) model, analogous considerations hold for the other models studied from first-principles or using GAP.

## Discussion

We have discussed a computational protocol that allows to determine from finite-size models of glasses containing less than 200 atoms—thus within the reach of standard first-principles approaches—the bulk limit of the harmonic Allen-Feldman conductivity^8,9^, as well as of the anharmonic Wigner conductivity^40,41^. To determine the bulk limit of the harmonic AF conductivity the following techniques are employed: (i) Fourier interpolation is used to improve the sampling of the vibrational spectrum of the glass model; (ii) the Dirac *δ* appearing in the AF conductivity is represented with a Gaussian broadening larger than the average vibrational energy-level spacing. The light-tailed Gaussian broadening is used in place of the originally proposed fat-tailed Lorentzian broadening^8,9^, to ensure that only quasi-degenerate velocity operator elements determine the thermal conductivity in the low-temperature harmonic limit, where anharmonicity phases out. To evaluate the bulk limit of the Wigner conductivity, the protocol uses a Voigt profile—a two-parameter distribution obtained as a convolution between the Gaussian used in the AF calculation, and the Lorentzian with FWHM determined by the linewidths appearing in the Wigner conductivity. The Voigt profile ensures that the effects of anharmonicity are considered only when they are not altered by finite-size effects (i.e., the linewidths are larger than the average energy-level spacings, and of the smallest broadening yielding computational convergence in an Allen-Feldman conductivity calculation). This allows to retain in finite-size models of glasses the physical property that heat transfer via a wave-like tunneling between neighboring (quasi-degenerate) vibrational eigenstates can occur even in the limit of vanishing anharmonicity, provided these eigenstates are not Anderson-localized, i.e., that they are coupled by non-zero velocity-operator elements.

The protocol has been validated on the paradigmatic glass *v*-SiO_2_, using a state-of-the-art GAP potential and atomistic models containing 192, 1536, or 5184 atoms^60^ to compute vibrational states; we have shown that employing the protocol on a 192-atom model allows to obtain harmonic (AF) and anharmonic (rWTE) conductivities in perfect agreement with those of the medium (1536-atom) and large (5184-atom) models generated using the same technique.

After validation, we have used the protocol to predict the AF and rWTE conductivities of *v*-SiO_2_ fully from first-principles. We have shown that anharmonicity does not significantly affect the conductivity of *v*-SiO_2_, even at high temperatures, since the AF conductivity is very similar to the rWTE conductivity over the entire temperature range analyzed (30 < *T* < 1300 K). We have supported this finding by showing that the rWTE conductivity, which accounts for anharmonicity at the lowest (third) perturbative order^41^, is compatible at high temperatures with the conductivity obtained from first-principles GKMD from Ercole et al.^26^ (we recall that GKMD simulations are accurate at high temperature, where the quantum specific heat approaches the classical limit and anharmonic effects are maximized). Our calculations are in agreement with experiments in the temperature range 50 ≲ *T* ≲ 450 K, but do not describe the surge of the conductivity observed at higher temperatures. Future work will aim at understanding if such a discrepancy can be related, as it seems likely, to radiative effects^105^.

The results obtained for *v*-SiO_2_ have allowed us to gain general insights on how anharmonicity affects the thermal conductivity of glasses at high temperature. Specifically, we have shown that the high-temperature trend of the conductivity is determined by how off-diagonal velocity-operator elements—which couple pairs of vibrational eigenstates (**q**)_*s*_ and ${({{{\bf{q}}}})}_{{s}^{{\prime} }}$, allowing tunnelling between them—vary as a function of the energy difference $\hslash {\omega }_{d}=\omega {({{{\bf{q}}}})}_{s}-\omega {({{{\bf{q}}}})}_{{s}^{{\prime} }}$. Velocity-operator elements increasing (decreasing) with ℏ*ω*_*d*_ drive a conductivity increase (decrease) with temperature. In the specific case of *v*-SiO_2_, the saturating trend of the conductivity derives from velocity-operator elements that are constant with respect to the energy difference between the eigenstates coupled.

Finally, we have interpreted heat conduction in terms of frequency-resolved thermal diffusivities for vibrations, showing that the harmonic Allen-Feldman diffusivity characterizes accurately thermal transport in vitreous silica at the microscopic level. This work paves the way to study the thermal conductivity of glasses from first principles (see e.g., ref. ^98^ for a recent application of this protocol to amorphous Al_2_O_3_ at various densities), and will be particularly relevant to investigate the thermal properties of amorphous materials for which developing quantum-accurate interatomic potentials is particularly challenging or unpractical.

## Methods

### Accuracy of the SMA approximation in glasses

**Fig. 9:**
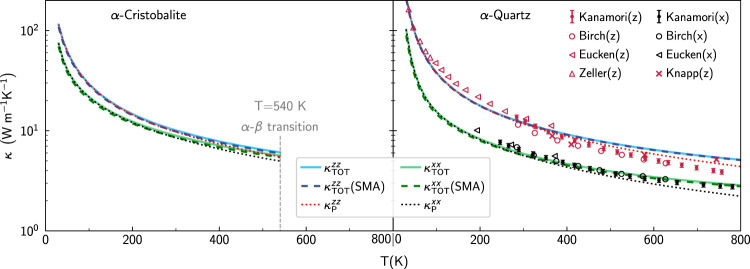
Thermal conductivity of crystalline *α*-cristobalite and *α*-quartz. For these materials the total bulk conductivity resulting from the full solution of the WTE (solid lines) is practically indistinguishable from the bulk conductivity computed relying on the SMA approximation (dashed lines). The *α*-*β* transition temperature for cristobalite is around 540 K^149^, below this temperature both *α*-cristobalite and *α*-quartz are to a good approximation simple crystals, since their total conductivity is predominantly determined by the particle-like contribution (*κ*_P_, dotted lines). Scatter points are experiments by Eucken^150^, Birch et al.^151^, Knapp^152^, Zeller et al.^96^ and Kanamori et al.^107^.

Glasses feature a low thermal conductivity, originating from strong scattering of vibrations due to anharmonicity or disorder. Past works^41,69,89,109^ have shown that as scattering becomes stronger, the relaxation to equilibrium of the vibrational excitations becomes faster, and therefore the SMA—which approximates the collision operator as in Eq. (4) (see ref. ^41^)—becomes more accurate. These considerations already suggest that the SMA approximation is accurate in vitreous silica. To further support these expectations, we investigated numerically the accuracy of the SMA approximation in the crystalline silica polymorphs *α*-cristobalite and *α*-quartz. These crystals have a conductivity much larger than vitreous silica, implying a weaker scattering from disorder or anharmonicity; consequently in *α*-cristobalite and *α*-quartz the SMA is expected^69,89,109^ to be less accurate than in vitreous silica. Figure 9 shows that for *α*-cristobalite and *α*-quartz solving the WTE in full or employing the SMA approximation yields conductivities practically indistinguishable; this demonstrates that the SMA is accurate in *α*-cristobalite and *α*-quartz, and also suggests that the SMA is even more accurate in vitreous silica.

### Effects of disorder and temperature on the linewidths

**Fig. 10:**
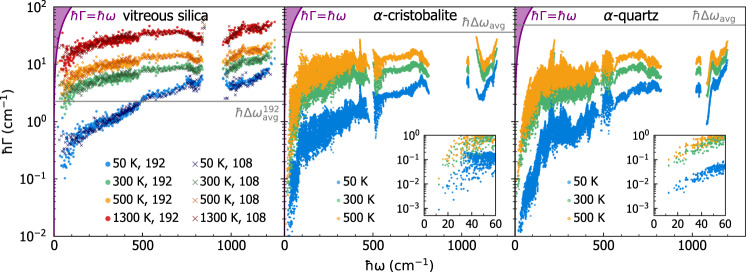
Effect of disorder and temperature on the linewidths of silica polymorphs. Increasing temperature yields an increase of the linewidths that account for third-order anharmonicity^88,89^ and isotopic-mass disorder^92^ in both vitreous and crystalline materials: the left panel shows the 192(D) and 108(D) vitreous-silica model (both computed from first-principles and at **q** = **0** only), the central panel shows *α*-cristobalite, and the right panel shows *α*-quartz. The temperatures of 50, 300, and 500 K at which the linewidths distributions are reported are chosen to span the temperature range over which all these materials are stable. The 1300 K distribution shows the behavior at high temperature, and is reported only for vitreous silica since *α*-cristobalite and *α*-quartz are not stable at this temperature. The insets in the central and right panels show the linewidths at low vibrational energies for *α*-cristobalite and *α*-quartz, respectively. The purple area shows the overdamped regime characterized by ℏΓ > ℏ*ω*; the lack of linewidths in the purple region shows that there are no overdamped vibrations and thus the Wigner formulation can be employed^41,47^. The gray lines represent the average spacing between the vibrational energy levels Eq. (6) (in the left panel only the average energy-level spacing for the 192-atom model is reported).

In Fig. 10 (left panel) we highlight how the frequency-linewidth distributions for models of vitreous silica having different size (192 or 108 atoms) are overlapping, suggesting that the linewidths of these models are not significantly affected by finite-size effects. In the central and right panels we show the linewidths of the crystalline polymorphs *α*-cristobalite (containing 12 atoms per primitive cell) *α*-quartz (9 atoms per primitive cell). Clearly, the variation of the anharmonic linewidths is mainly due to temperature, since at fixed temperature the frequency-linewidth distributions of amorphous and crystalline silica polymorphs have a similar magnitude. We also note that the anharmonic linewidths computed with GAP for the 192(G) model (Fig. 3) are very similar to the anharmonic linewidths computed from first-principles for the 192(D) and 108(D) models (Fig. 10 left panel).

### Accounting for anharmonicity at a reduced computational cost

In this section we discuss the details of the computation of the analytical function Γ_a_[*ω*], used to approximatively determine the linewidths as a function of frequency discussed in Fig. 3. The analytical function Γ_a_[*ω*] is determined as

10 $${\Gamma }_{{{{\rm{a}}}}}[\omega ]=\frac{1}{\sqrt{\frac{1}{{({\Gamma }_{1}[\omega ])}^{2}}+\frac{1}{{({\Gamma }_{2}[\omega ])}^{2}}}},$$

where Γ_1_[*ω*] and Γ_2_[*ω*] are defined as

11 $$\begin{array}{lll}{\Gamma }_{1}[\omega ]\,=\,\frac{{\sum }_{{{{\bf{q}}}} = {{{\bf{0}}}},s}\frac{1}{\sqrt{2\pi {\sigma }^{2}}}\exp \left[-\frac{{\hslash }^{2}{(\omega {({{{\bf{q}}}})}_{s}-\omega )}^{2}}{2{\sigma }^{2}}\right]}{{\sum }_{{{{\bf{q}}}} = {{{\bf{0}}}},s}\frac{1}{\Gamma{({{{\bf{q}}}})}_{s}}\frac{1}{\sqrt{2\pi {\sigma }^{2}}}\exp \left[-\frac{{\hslash }^{2}{(\omega {({{{\bf{q}}}})}_{s}-\omega )}^{2}}{2{\sigma }^{2}}\right]},\\ {\Gamma }_{2}[\omega ]\,=\,p\cdot {\omega }^{2},\\ \qquad p\,=\,\frac{{\sum }_{{{{\bf{q}}}} = {{{\bf{0}}}},s}\int\nolimits_{{\omega }_{{{{\rm{o}}}}}}^{2{\omega }_{{{{\rm{o}}}}}}d{\omega }_{c}\frac{\Gamma {({{{\bf{q}}}})}_{s}}{{\omega }^{2}{({{{\bf{q}}}})}_{s}}\frac{1}{\sqrt{2\pi {\sigma }^{2}}}\exp \left[-\frac{{\hslash }^{2}{(\omega {({{{\bf{q}}}})}_{s}-{\omega }_{c})}^{2}}{2{\sigma }^{2}}\right]}{{\sum }_{{{{\bf{q}}}} = {{{\bf{0}}}},s}\int\nolimits_{{\omega }_{{{{\rm{o}}}}}}^{2{\omega }_{{{{\rm{o}}}}}}d{\omega }_{c}\frac{1}{\sqrt{2\pi {\sigma }^{2}}}\exp \left[-\frac{{\hslash }^{2}{(\omega {({{{\bf{q}}}})}_{s}-{\omega }_{c})}^{2}}{2{\sigma }^{2}}\right]}.\end{array}$$

**Fig. 11:**
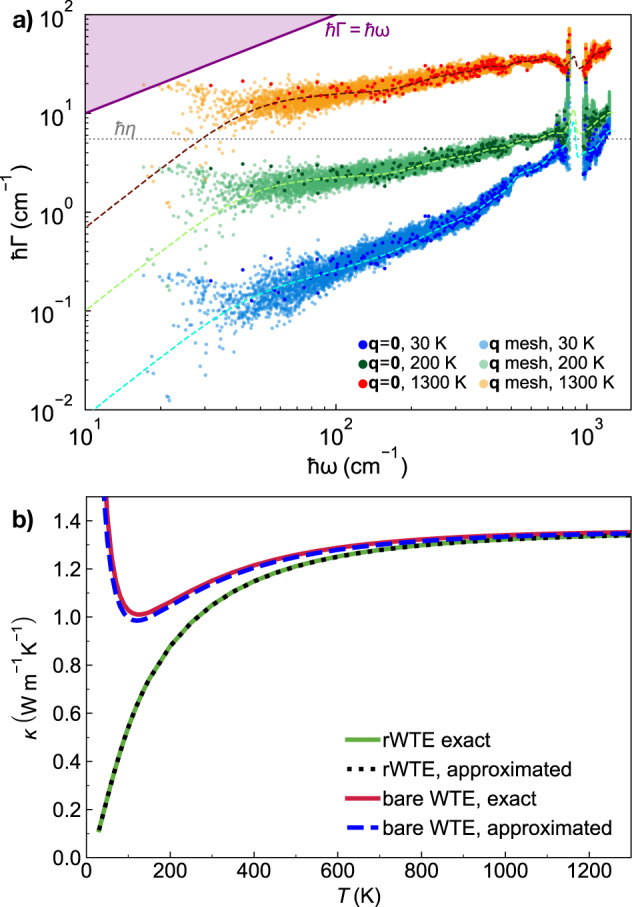
Effect of **q** interpolation on the linewidths and approximation to reduce the computational cost. **a** Linewidths of the 108(D) model of *v*-SiO_2_ computed explicitly on a 5 × 5 × 5 **q** mesh (light blue is at 30 K, light green is at 200 K, and orange is at 1300 K) or computed at the point **q** = **0** only (dark blue is at 30 K, dark green is at 200 K, and red is at 1300 K). The purple region represents the overdamped regime, where vibrations cannot be accurately described using the Wigner formulation and spectral-function approaches have to be employed^41,47^. The horizontal dotted line is the broadening ℏ*η* used in the Voigt renormalization for the 108-atom model, all the linewidths below this line are regularized and thus have negligible effect on the rWTE conductivity. The dashed lines are the analytical functions Γ_a_[*ω*], determined from the distributions at **q** = **0** only as detailed in Eqs. (10), (11). **b** Shows the bare WTE conductivity of the 108(D) model computed exactly (i.e., using the linewidths explicitly computed on the 5 × 5 × 5 **q** mesh, solid red), or using the linewidths approximatively determined using the function Γ_a_[*ω*] (dashed blue); the solid green and dotted black lines show the rWTE conductivities computed using the exact or approximated linewidths, respectively.

*ω*_o_ is the smallest non-zero frequency at **q** = **0** and *σ* = 30 cm^−1^ is a broadening chosen sufficiently large to ensure that the linewidths are averaged in a smooth way. The functional form of the approximated function Γ_a_[*ω*] is inspired by past work^51,93^, and the specific expressions (10), (11) to determine it have been devised and validated relying on exact calculations performed on the 108(D) *v*-SiO_2_ model. Specifically, we show in Fig. 11a that the approximated functions Γ_a_[*ω*] (dashed lines, different colors show different temperatures) captures the trend of the linewidths explicitly computed over a dense 5 × 5 × 5 **q** mesh (dense distributions). We recall that Γ_a_[*ω*] is determined from the linewidth distributions at **q** = **0** only (coarse clouds of scatter points). In Fig. 11b we show that computing the bare WTE conductivity over a dense 5 × 5 × 5 **q** mesh using the anharmonic linewidths computed exactly (solid red) or determined approximatively using the function Γ_a_[*ω*] (dashed blue) are practically indistinguishable; consequently, also the rWTE conductivity computed over the same 5 × 5 × 5 **q** mesh is practically unchanged when the exact (solid green) or approximated (dotted black) linewidths are used. The good agreement between the exact and the approximated calculations shows that the analytical function Γ_a_[*ω*] allows to account for anharmonicity at a reduced computational cost and without appreciably compromising accuracy, both in the WTE and rWTE calculations.

### Finite-size effects

**Fig. 12:**
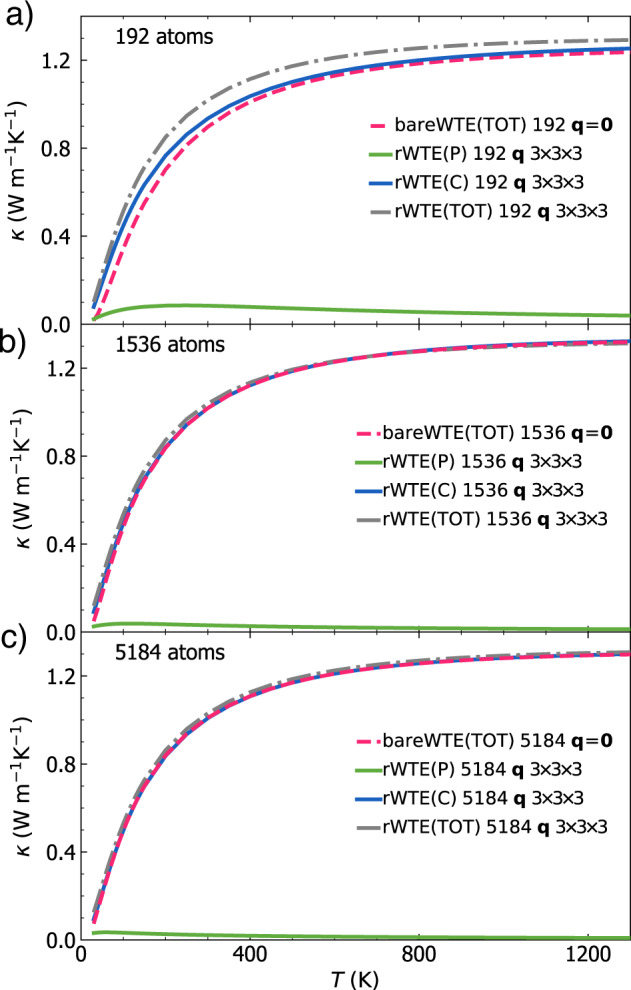
Decomposition of the total thermal conductivity of finite-size models of *v*-SiO_2_ into populations and coherences terms. **a** Results for the small `192(G)' model. The dashed red line is the bare WTE conductivity computed at **q** = **0**; the lack of perfectly degenerate vibrational modes in this disordered model and zero diagonal elements ($s={s}^{{\prime} }$) of the velocity operator at **q** = **0** implies that the total bare WTE conductivity originates entirely from coherences. The solid green and blue lines are the populations and coherences rWTE conductivities computed on a 3 × 3 × 3 **q** mesh, their sum is the bulk limit of the total rWTE conductivity (dashed-dotted gray). **b** and **c**: results for the medium 1536(G) and large 5184(G) models, styles are the same as before. We highlight how the populations conductivity (green) decreases as the size of the model increases, such a decrease is compensated by an increase of the coherences conductivity; this implies that these different models have practically indistinguishable total thermal conductivities. In practice, in the 1536(G) and 5184(G) models the bare WTE computed at **q** = **0** reproduces the total rWTE conductivity.

As anticipated in Sec. *Extension of the protocol to evaluate the anharmonic Wigner conductivity*, in a finite-size model of a glass the distinction between populations and coherences conductivities (see Sec. *Wigner formulation of thermal transport*) is useful to understand finite-size effect, but cannot be further interpreted in terms of particle-like and wave-like conduction mechanisms. In fact, such an interpretation is well defined only in crystals^41^ where the BZ and vibrational spectrum can be unambiguously defined^68^, while in finite-size models of glasses the BZ volume decreases as the size of the model increases. Keeping these considerations in mind, we show in Fig. 12 how the populations and coherences rWTE conductivities depend on the size of the atomistic model. It is evident that increasing the size of the model’s reference cell yields decrease in the ratio between the populations and coherences conductivities, leaving their sum approximately constant. This implies that the bare WTE conductivity evaluated at **q** = **0** only—which in disordered systems without perfectly degenerate vibrational modes entirely originates from coherences—becomes an increasingly more accurate estimate for the total conductivity as the size of the model’s reference cell increases. We highlight how in the small 192-atom model the populations conductivity computed on the **q** mesh is non-negligible and has to be considered to correctly describe the bulk limit (rWTE).

To compute the conductivities in Fig. 12, the anharmonic linewidths have been determined using the analytical function Γ_a_[*ω*] shown in Fig. 3, broadenings *η* equal to 4.0, 1.0, and 0.3 cm^−1^ (around the beginning of the conductivity plateaus shown in Fig. 2) have been used for the Voigt profile for the 192-, 1536-, and 5184-atom models, respectively.

### Quantum harmonic specific heat

**Fig. 13:**
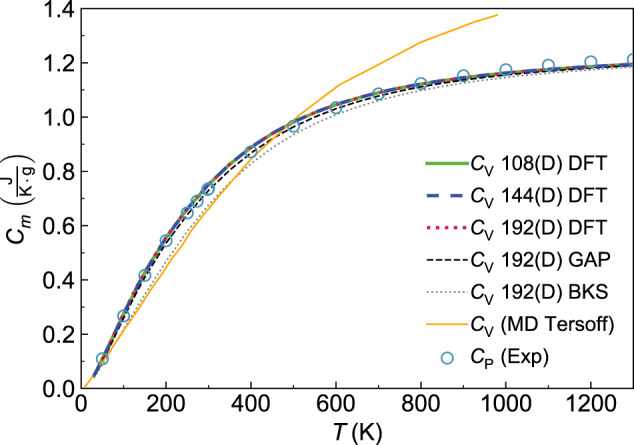
Specific heat of vitreous silica. Thick lines are the quantum harmonic specific heat at constant volume (*C*_V_) and as a function of temperature computed from first principles for the 108(D) (green), 144(D) (blue) and 192(D) (red) vitreous-silica models. The thin dashed-black (dotted-gray) line is *C*_V_ for the 192(D) model computed using the GAP (BKS) potential. The solid orange line is *C*_V_ of amorphous silica computed from constant-volume molecular dynamics simulations using the Tersoff potential, taken from ref. ^24^. Circles are experimental measurements^110^ of the specific heat at constant pressure.

We show in Fig. 13 that the theoretical quantum harmonic specific heat at constant volume (${C}_{{{{\rm{V}}}}}^{{{{\rm{Th}}}}}(T)=\frac{1}{\rho {{{\mathcal{V}}}}{N}_{{{{\rm{c}}}}}}{\sum }_{{{{\bf{q}}}},s}C{({{{\bf{q}}}})}_{s}$, where *ρ* is the density) is in close agreement with the experimental specific heat at constant pressure^110^. This suggests that the renormalization of vibrational energies due to anharmonicity and temperature^45,53,111,112^ are negligibly small for *v*-SiO_2_ in the temperature range considered^113^, and as such these effects are not considered in this work. Figure 13 also shows that first-principles and GAP calculations yield more precise estimates of the specific heat compared to the BKS and Tersoff potentials (the latter has been recently employed in ref. ^114^ to study the thermal properties of *v*-SiO_2_).

### Velocity operator as a function of frequency

In this section we provide all the details on the plot reported in Fig. 7. In order to gain insights into the trend of the conductivity with temperature (Fig. 5), we recast the conductivity expression (1) as a function of ${\omega }_{{{{\rm{a}}}}}=(\omega {({{{\bf{q}}}})}_{s}+\omega {({{{\bf{q}}}})}_{{s}^{{\prime} }})/2$ and ${\omega }_{{{{\rm{d}}}}}=\omega {({{{\bf{q}}}})}_{s}-\omega {({{{\bf{q}}}})}_{{s}^{{\prime} }}$:

12 $$\begin{array}{lll}\kappa \,=\,\int\limits_{0}^{{\omega }_{\max }}d{\omega }_{{{{\rm{a}}}}}\int\limits_{-{\omega }_{\max }}^{{\omega }_{\max }}d{\omega }_{{{{\rm{d}}}}}\left[\frac{1}{{{{\mathcal{V}}}}{N}_{{{{\rm{c}}}}}}\mathop{\sum}\limits_{{{{\bf{q}}}},s,{s}^{{\prime} }}\frac{\omega {({{{\bf{q}}}})}_{s}+\omega {({{{\bf{q}}}})}_{{s}^{{\prime} }}}{4}\frac{\parallel {{\mathbf{v}}}{({{{\bf{q}}}})}_{s,{s}^{{\prime} }}{\parallel }^{2}}{3}\right.\\ \qquad\times \,\left(\frac{C{({{{\bf{q}}}})}_{s}}{\omega {({{{\bf{q}}}})}_{s}}+\frac{C{({{{\bf{q}}}})}_{{s}^{{\prime} }}}{\omega {({{{\bf{q}}}})}_{{s}^{{\prime} }}}\right)\pi {{{{\mathcal{F}}}}}_{[\Gamma {({{{\bf{q}}}})}_{s}+\Gamma {({{{\bf{q}}}})}_{{s}^{{\prime} }},\eta ]}(\omega {({{{\bf{q}}}})}_{s}-\omega {({{{\bf{q}}}})}_{{s}^{{\prime} }})\\ \qquad\times \,\left.{\delta }_{{\sigma }_{{{{\rm{a}}}}}}\left(\frac{\omega {({{{\bf{q}}}})}_{s}+\omega {({{{\bf{q}}}})}_{{s}^{{\prime} }}}{2}-{\omega }_{{{{\rm{a}}}}}\right){\delta }_{{\sigma }_{{{{\rm{d}}}}}}\left((\omega {({{{\bf{q}}}})}_{s}-\omega {({{{\bf{q}}}})}_{{s}^{{\prime} }})-{\omega }_{{{{\rm{d}}}}}\right)\right]\end{array}$$

where the distributions *δ*_*σ*_ are Gaussian broadenings of the Dirac delta:

13 $${\delta }_{\sigma }\left(\Omega -\omega \right)=\frac{1}{\sqrt{2\pi }\sigma }\exp \left[-\frac{1}{2{\sigma }^{2}}{\left(\Omega -\omega \right)}^{2}\right].$$

In order to achieve our goal to recast Eq. (12) in terms of physically insightful frequency-dependent functions, we approximate the linewidths using the single-valued function of frequency, i.e., Γ(**q**)_*s*_ = Γ_a_[*ω*(**q**)_*s*_] (see Eq. (10)); this approximation allows to recast Eq. (12) as follows:

14 $$\begin{array}{lll}\kappa \,=\,\int\limits_{0}^{{\omega }_{\max }}d{\omega }_{{{{\rm{a}}}}}\int\limits_{-{\omega }_{\max }}^{{\omega }_{\max }}d{\omega }_{{{{\rm{d}}}}}{{{\mathcal{G}}}}({\omega }_{{{{\rm{a}}}}},{\omega }_{{{{\rm{d}}}}}){{{\mathcal{C}}}}({\omega }_{{{{\rm{a}}}}},{\omega }_{{{{\rm{d}}}}})\left\langle | \sf{v}_{{\omega }_{{{{\rm{a}}}}},{\omega }_{{{{\rm{d}}}}}}^{{{{\rm{avg}}}}}{| }^{2}\right\rangle \\ \qquad\times \,\pi {{{{\mathcal{F}}}}}_{[{\Gamma }_{{{{\rm{a}}}}}[{\omega }_{{{{\rm{a}}}}}+\frac{{\omega }_{{{{\rm{d}}}}}}{2}]+{\Gamma }_{{{{\rm{a}}}}}[{\omega }_{{{{\rm{a}}}}}-\frac{{\omega }_{{{{\rm{d}}}}}}{2}],\eta ]}({\omega }_{{{{\rm{d}}}}}),\end{array}$$

where ${{{\mathcal{G}}}}({\omega }_{{{{\rm{a}}}}},{\omega }_{{{{\rm{d}}}}})$ is a density of states

15 $$\begin{array}{lll}{{{\mathcal{G}}}}({\omega }_{{{{\rm{a}}}}},{\omega }_{{{{\rm{d}}}}})\,=\,\frac{1}{{N}_{{{{\rm{at}}}}}}\frac{1}{{{{\mathcal{V}}}}{N}_{{{{\rm{c}}}}}}\mathop{\sum}\limits_{{{{\bf{q}}}},s,{s}^{{\prime} }}{\delta }_{{\sigma }_{{{{\rm{a}}}}}}\left(\frac{\omega {({{{\bf{q}}}})}_{s}+\omega {({{{\bf{q}}}})}_{{s}^{{\prime} }}}{2}-{\omega }_{{{{\rm{a}}}}}\right)\\ \qquad\qquad\qquad\times \,{\delta }_{{\sigma }_{{{{\rm{d}}}}}}\left((\omega {({{{\bf{q}}}})}_{s}-\omega {({{{\bf{q}}}})}_{{s}^{{\prime} }})-{\omega }_{{{{\rm{d}}}}}\right),\end{array}$$

${{{\mathcal{C}}}}({\omega }_{{{{\rm{a}}}}},{\omega }_{{{{\rm{d}}}}})$ is a specific heat

16 $${{{\mathcal{C}}}}({\omega }_{{{{\rm{a}}}}},{\omega }_{{{{\rm{d}}}}})=\frac{{\omega }_{{{{\rm{a}}}}}}{2}\left(\frac{C({\omega }_{{{{\rm{a}}}}}+\frac{{\omega }_{{{{\rm{d}}}}}}{2})}{{\omega }_{{{{\rm{a}}}}}+\frac{{\omega }_{{{{\rm{d}}}}}}{2}}+\frac{C({\omega }_{{{{\rm{a}}}}}-\frac{{\omega }_{{{{\rm{d}}}}}}{2})}{{\omega }_{{{{\rm{a}}}}}-\frac{{\omega }_{{{{\rm{d}}}}}}{2}}\right),$$

and $\langle | \sf{v}_{{\omega }_{{{{\rm{a}}}}},{\omega }_{{{{\rm{d}}}}}}^{{{{\rm{avg}}}}}{| }^{2}\rangle$ is the average square modulus of the velocity operator defined in Eq. (7) (whose Dirac delta must be broadened with the Gaussian *δ*_*σ*_ discussed here) and plotted in Fig. 7. Eq. (14), together with Fig. 7 and Fig. 10, sheds light on the saturating trend of the conductivity with temperature (Fig. 5a)). In fact, among the various quantities entering in Eq. (14), the density of states (15) and the specific heat (16) have a trivial temperature dependence (the former is temperature-independent, the latter saturates with increasing temperature). The change of variable performed in Eq. (12) shows that the temperature-conductivity trend is determined by how the average square velocity-operator elements vary with the vibrational frequency difference *ω*_d_, because the increase of the linewidths with temperature (Fig. 10a)) results in a broader Lorentzian distribution (4) that encloses velocity-operator elements corresponding to increasingly larger frequency differences *ω*_d_. In particular, for vitreous silica, the average square velocity-operator elements are almost independent from *ω*_d_ for all values of *ω*_a_ (Fig. 7). It follows that the saturating trend of the conductivity at high temperature reported in Fig. 5a) is inherited from the saturating trend of the specific heat.

It is also worth noting that in the harmonic AF limit (ℏ*η* ≳ Δ*ω*_avg_ ≫ ℏΓ(**q**)_*s*_ → 0 ∀ **q**, *s*) the Voigt profile reduces to the Gaussian representation of the Dirac delta and consequently only the quasi-degenerate velocity-operator elements ($\mathop{\lim }\nolimits_{{\omega }_{{{{\rm{d}}}}}\to 0}\langle | \sf{v}_{{\omega }_{{{{\rm{a}}}}},{\omega }_{{{{\rm{d}}}}}}^{{{{\rm{avg}}}}}{| }^{2}\rangle$) contribute to the conductivity. To see this, it is sufficient to replace the Voigt profile in Eq. (14) with the Dirac delta, obtaining:

17 $$\kappa =\int\limits_{0}^{{\omega }_{\max }}d{\omega }_{{{{\rm{a}}}}}C({\omega }_{{{{\rm{a}}}}}){{{\mathcal{G}}}}({\omega }_{{{{\rm{a}}}}},0)\int\limits_{-{\omega }_{\max }}^{{\omega }_{\max }}d{\omega }_{{{{\rm{d}}}}}\left\langle | \sf{v}_{{\omega }_{{{{\rm{a}}}}},{\omega }_{{{{\rm{d}}}}}}^{{{{\rm{avg}}}}}{| }^{2}\right\rangle \pi \delta ({\omega }_{{{{\rm{d}}}}}),$$

where *C*(*ω*_a_) is the specific heat defined in Eq. (2) and ${{{\mathcal{G}}}}({\omega }_{{{{\rm{a}}}}},0)$ is obtained from Eq. (15) in the limit *ω*_d_ → 0.

In summary, we have shown how the harmonic Allen-Feldman conductivity is determined exclusively by the quasi-degenerate velocity operator elements ($\mathop{\lim }\nolimits_{{\omega }_{{{{\rm{d}}}}}\to 0}\langle | \sf{v}_{{\omega }_{{{{\rm{a}}}}},{\omega }_{{{{\rm{d}}}}}}^{{{{\rm{avg}}}}}{| }^{2}\rangle$), while the anharmonic Wigner conductivity receives contributions from velocity-operator elements having *ω*_d_ spanning the entire frequency range.

Figure 7 is computed relying on the 192(D) model and on first-principles calculations, using a 3 × 3 × 3 **q** point mesh, ℏ*σ*_a_ = 15 cm^−1^ and $\hslash {\sigma }_{{{{\rm{d}}}}}=\sqrt{\frac{\pi }{2}}\hslash \eta =5$ cm^−1^ (this latter corresponds to a Gaussian having height $\frac{1}{\pi \eta }$, with ℏ*η* = 4 cm^−1^ equal to the one used in the computation of the AF conductivity for the 192(D) model). Increasing the **q** point mesh to 5 × 5 × 5 or multiplying *σ*_a_ and *σ*_d_ by a factor of 2 does not produce significant changes. We have verified that plotting the velocity operator for the other models studied from first principles or using GAP yields results that are practically indistinguishable from those reported in Fig. 7.

### Effects of anharmonicity using the BKS potential

**Fig. 14:**
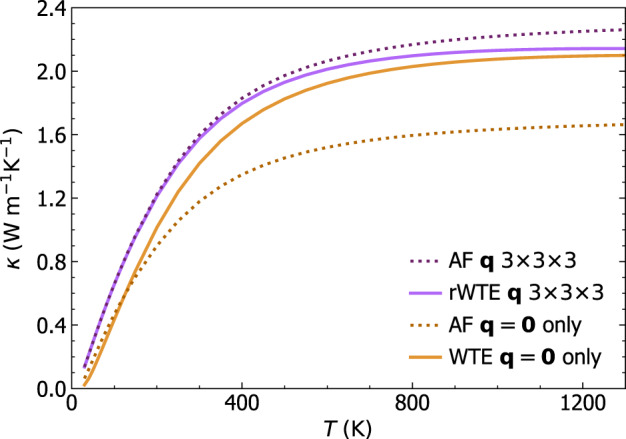
Negligible effects of anharmonicity in *v*-SiO_2_ described with the BKS potential. Evaluating the conductivity using the protocol to determine the bulk limit yields an anharmonic rWTE conductivity (solid purple) that differs only about 4% from the bulk limit of the AF conductivity (dotted purple, evaluated using a Gaussian broadening ℏ*η* = 4 cm^−1^ determined from a convergence test analogous to that reported in Fig. 2). In contrast, evaluating the AF and WTE conductivities at **q** = **0** only (and using a Lorentzian broadening ℏ*η* = 1.6 cm^−1^ in the former case) yield values that strongly underestimates the bulk value for the AF conductivity (dotted orange) and weakly underestimates the Wigner conductivity (solid orange), respectively.

While our BKS-based rWTE predictions are in agreement with ref. ^84^, our BKS-based AF conductivity is significantly different from that reported in ref. ^84^, which claimed that anharmonicity enhances appreciably the conductivity in *v*-SiO_2_ (specifically, ref. ^84^ concluded that in *v*-SiO_2_ in the high-temperature limit, the anharmonic WTE conductivity is about 30% higher than the AF conductivity). In Fig. 14 we show that evaluating the AF conductivity as done in ref. ^84^, i.e., using the BKS potential, at **q** = **0** only and using a Lorentzian broadening ℏ*η* = 1.6 cm^−1^, yields a value that significantly underestimates the bulk value for the AF conductivity. In contrast, determining the bulk limit of the AF conductivity using the protocol discussed here, i.e., the Fourier interpolation with a computationally converged broadening ℏ*η* = 4 cm^−1^, yields a larger AF conductivity, which differs only about 4% from the bulk limit of the rWTE conductivity.

### Computational details

#### First-principles calculations

All the density-functional theory (DFT) calculations have been performed with the Quantum ESPRESSO distribution^115^ using the PBE functional with Grimme-D2 corrections (PBE+D2)^116^. This choice is motivated by the benchmarks given in ref. ^117^ and accounting for the capability of Quantum ESPRESSO to compute phonons using density-functional perturbation theory (DFPT)^118^ with the PBE+D2 exchange-correlation functional. This choice is validated by the agreement between theoretical and experimental densities reported in Table 1 and also by the capability of this functional to accurately describe the thermal properties of *α*-quartz (Fig. 9). We have used pseudopotentials from the SSSP efficiency library^119,120^ with a cutoff of 50 Ry and a dual of 8. In the following we report the details for all the three different systems studied: vitreous silica, *α*-cristobalite, and *α*-quartz.

**Table 1:** Densities of silica polymorphs.

Structure	*ρ* (k*g*/*m*^3^)
*v*-SiO_2_, 192(D) PBE+D2 (SiO2 2818^65^)	2241.2
*v*-SiO_2_, 192(D) GAP (SiO2 2818^65^)	2288.6
*v*-SiO_2_, 192(D) BKS (SiO2 2818^65^)	2243.8
*v*-SiO_2_, 192(G) GAP (*#*4^60^)	2188.7
*v*-SiO_2_, 1536(G) GAP (this work)	2257.1
*v*-SiO_2_, 5184(G) GAP^60^	2203.6
*v*-SiO_2_, 144(D) PBE+D2^63^	2220.6
*v*-SiO_2_, 108(D) PBE+D2 (SiO2.1586^62^)	2243.9
*v*-SiO_2_, Experiment^138^	2203 ± 3
*v*-SiO_2_, Experiment^139^	2200 ± 10
*α*-cristobalite^129^, PBE+D2	2383.5
*α*-cristobalite, Experiment^140^	2326 ± 12
*α*-quartz^130^, PBE+D2	2641.9
*α*-quartz, Experiment^141^	2650

Comparison between theoretical and experimental densities *ρ* for the various silica polymorphs analyzed.

**Fig. 15:**
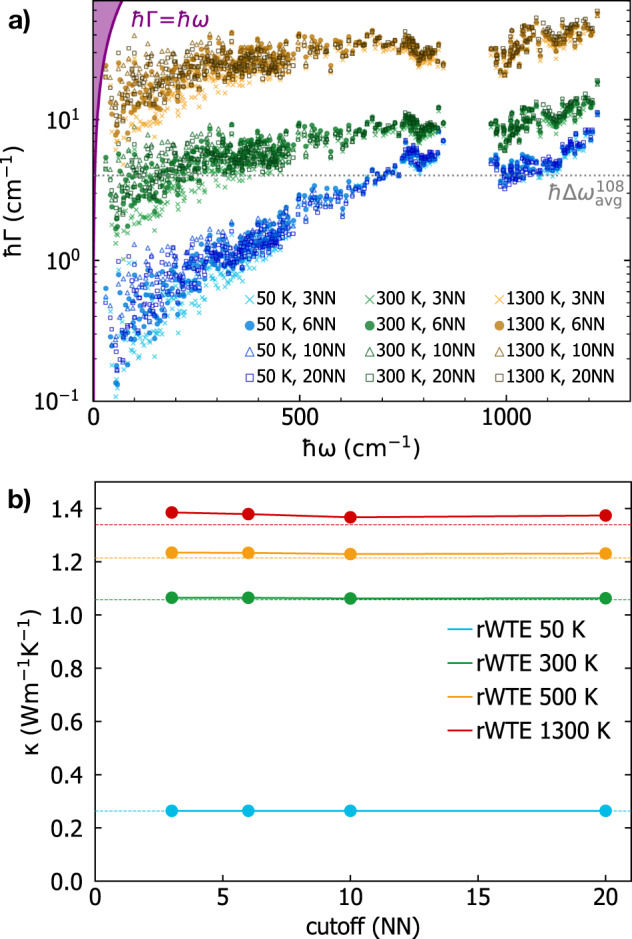
Convergence of linewidths and rWTE conductivity with respect to cutoff for third-order IFC. **a** Linewidth distribution for the 108(D) model at **q** = **0** and using different cutoffs: crosses are 3rd nearest neighbor (0.26 nm, 12348 displacements), circles are 6th nearest neighbor (0.32 nm, 18972 displacements), triangles are 10th nearest neighbor (0.39 nm, 29592 displacements), squares are 20th nearest neighbor (0.46 nm, 53352 displacements). **b** Solid lines are the rWTE conductivities at different temperatures computed using the function Γ_a_[*ω*] determined from the linewidths distributions in (**a**) using Eqs. (10), (11); the dashed lines are the Allen-Feldman conductivities.

*Vitreous silica*. The 108(D) structure of vitreous silica is taken from ref. ^62^ (‘SiO2.1586’ model). For this structure, cell parameters and atomic positions are relaxed with DFT using Γ-point (**q** = **0**) sampling, a threshold of 10^−4^ Ry/Bohr for the atomic forces (i.e., a structure is considered relaxed if all the Cartesian components of the forces acting on atoms are smaller than this threshold), and a threshold of 0.1 kBar for pressure. The harmonic dynamical matrices (which yield the vibrational frequencies and velocity operators) are computed using DFPT on a 2 × 2 × 2 **q** point mesh and accounting for the non-analytic term correction due to the dielectric tensor and Born effective charges. Third-order interatomic force constants (IFC) are computed in the reference cell using ShengBTE^89^, and with a cutoff of 0.32 nm (corresponding to the 6th nearest neighbor (NN)). We show in Fig. 15 that increasing this cutoff to the 10th and 20th NN does not yield significant changes to the final results (linewidth distributions and rWTE conductivities). The third-order IFC are converted from ShengBTE to Phono3py^91^ format using hiphive^121^ (we adopted this procedure for software compatibility reasons, since when we started this study the ShengBTE format was the one interfaced with the largest number of transport codes: AlmaBTE^90^, thermal2^87,88,122^, Phoebe^123^, ALAMODE^124^, and, as mentioned before, Phono3py^91^ through hiphive^121^).

The linewidths are computed using Phono3py^91^ on a 5 × 5 × 5 **q** point mesh using the tetrahedron method, we checked that computing them using Gaussian smearing of ℏ*σ* = 2 cm^−1^ for the Dirac delta appearing in the linewidth expression (see e.g., Eq. (11) of ref. ^91^) does not produce appreciable changes. Thermal conductivity calculations are performed using a **q** interpolation mesh equal to 5 × 5 × 5. The Voigt profile, used to combine the AF and the WTE conductivities as detailed before, has been numerically implemented following the prescriptions reported in ref. ^94^ and summarized in Sec. *Implementation of the Voigt profile*. We have verified that reducing the **q** interpolation mesh to 3 × 3 × 3 does not produce appreciable changes.

The 192(D) model is generated using the same techniques of ref. ^62^ and is discussed in ref. ^65^, while the 144(D) model is taken from ref. ^63^ and is available in the Materials Cloud Archive^64^. For all these structures, the relaxation of the reference cell with DFT, the calculation of the harmonic dynamical matrices, and the the calculation of third-order anharmonic force constants are performed using the same parameters used for the 108-atom structure. These harmonic and anharmonic force constants are then used to compute the linewidths at the point **q** = **0** only using Phono3py^91^ and a Gaussian smearing of 2 cm^−1^, and then employed within the scheme discussed and validated in Fig. 11. We also verified that the 192(D) model accurately reproduces the experimental bulk modulus of *v*-SiO_2_ (the theoretical value we computed is 36.9 GPa, while the experimental value of ref. ^125^ is 36.8 GPa and the experimental value of ref. ^126^ is 36.9 GPa).

An analysis of the coordination numbers using the procedure based on the minimum of the radial distribution function as implemented in the R.I.N.G.S. software^127^ or that based on the position of the Wannier centers discussed in ref. ^128^ have revealed in both cases that the 108(D), 144(D), 192(D) vitreous structures considered in this work do not have coordination defects or lone pairs (both before and after the DFT relaxation).

*α*-*cristobalite*. The crystal structure of *α*-cristobalite is taken from ref. ^129^ (ICSD collection code 47219). In first-principles calculations, the Brillouin zone is integrated with a Monkhorst-Pack mesh of 5 × 5 × 4 points, with a (1, 1, 1) shift. Second-order force constants are computed using DFPT on a 5 × 5 × 4 **q** mesh, accounting also for the non-analytic term correction due to the dielectric tensor and Born effective charges. To obtain the third-order IFC the finite-difference method implemented in ShengBTE^89^ is used, together with the interconversion software from ShengBTE to Quantum ESPRESSO, available in the thermal2 package^69,87^. In these third-order force constants calculations, a 2 × 2 × 2 supercell with a 2 × 2 × 2 k-point sampling is used, and interactions up to the 6th nearest neighbor (corresponding to ~ 0.39 nm) are considered. We have checked that running the same thermal conductivity calculation in the Phono3py software yielded compatible results. The linewidths (Fig. 10b) and thermal conductivity (Fig. 9a) are computed with the thermal2 package^69,87^ using a 17 × 17 × 13 **q**-point mesh and a Gaussian smearing ℏ*σ* = 4 cm^−1^.

*α*-*quartz* The crystal structure of *α*-quartz is taken from ref. ^130^ (Crystallographic Open Database id 1526860). In first-principles calculations, the Brillouin zone is integrated with a Monkhorst-Pack mesh of 6 × 6 × 5 points, with a (1, 1, 1) shift. Second-order force constants are computed using DFPT on a 4 × 4 × 4 **q** mesh, accounting also for the non-analytic term correction due to the dielectric tensor and Born effective charges. Third-order force constants are computed using the finite-difference methods as implemented in ShengBTE^89^, using a 3 × 3 × 2 supercell with a Γ-only k-point sampling, and a cutoff for atomic interactions of 0.9 nm. The linewidths (Fig. 10c) and thermal conductivity (Fig. 9b)) are computed with the thermal2 package^69,87^ using a 19 × 19 × 15 **q**-point mesh and a Gaussian smearing ℏ*σ* = 4 cm^−1^. We have checked that running the same thermal conductivity calculation in the Phono3py software yielded compatible results. Our results are also compatible with those reported in ref. ^131^.

#### Calculations performed using the GAP potential

The GAP potential for vitreous silica was taken from ref. ^60^. The 5184(G) and 192(G) models discussed in Figs. 1–4 are taken from ref. ^60^, we chose the 192-atom structure number 4 in that reference as the one having the closest density to the large 5184-atom model. More details on these structures are reported in ref. ^60^.

The 1536(G) model was generated starting from a 2 × 2 × 2 supercell of the 192(G) model, thus using the melt-quench protocol of ref. ^60^. Specifically, we heated the structure to 6000 K (NVT), equilibrated it for 10 ps (NVT), cooled it to 4000 K in 10 ps (NVT), equilibrated the melt at 4000 K for 100 ps (NPT), thus quenched the structure to 1 K with a quench rate 10^13^ K/s (NPT). The calculation was performed with LAMMPS^132^, using the quip^133–135^ interface to call the GAP potential routines. A timestep of 0.001 ps was used, and generating this structure required about 12 kCPUh (1 kCPUh = 1000 CPU core hours) on the Icelake nodes of the Cambridge Service for Data-Driven Discovery. The densities of the models analyzed are reported in Table 1.

Interatomic forces and stress tensor are computed using LAMMPS^132^, using the quip^133–135^ interface to call the GAP potential routines. Cell parameters and atomic positions are relaxed using a threshold of 25 eV/Angstrom for the atomic forces (i.e., a structure is considered relaxed if all the Cartesian components of the forces acting on atoms are smaller than this threshold), and a threshold of 0.1 kBar for pressure. The harmonic dynamical matrices (which yield the vibrational frequencies and velocity operators) are computed using Phonopy, on a 2 × 2 × 2 supercell for the 192(G) structure, and on the reference cell (1 × 1 × 1) for the 1536(G) and 5184(G) models. For the 192(G) model, third-order force constants are computed in the reference cell using ShengBTE^89^, using a cutoff equal to the 6th nearest-neighbor; the resulting force constants are then converted in Phono3py^91^ format using hiphive^121^. The anharmonic linewidths for the 192(G) model (shown in Fig. 3) are computed using Phono3py at **q** = **0** and using Gaussian smearing of ℏ*σ* = 0.4 cm^−1^ for the Dirac delta appearing in the expression for the linewidths (Eq. (11) of ref. ^91^). We have checked that using a larger Gaussian broadening ℏ*σ* = 2 cm^−1^ yields linewidth distributions that overlaps with those reported in Fig. 3, and yield a rWTE conductivities with a relative difference ($\frac{{\kappa }_{{{{\rm{rWTE}}}}}^{0.4\,{{{{\rm{cm}}}}}^{-1}}-{\kappa }_{{{{\rm{rWTE}}}}}^{2\,{{{{\rm{cm}}}}}^{-1}}}{{\kappa }_{{{{\rm{rWTE}}}}}^{2\,{{{{\rm{cm}}}}}^{-1}}}\cdot 100$) always smaller than 0.51%. We note in passing that the broad range of values assumed by the linewidths distributions of Fig. 3 in the low-energy limit (in practice in the range 30–40 cm^−1^) is compatible with that observed in *α*-cristobalite and *α*-quartz in the same frequency range (see Fig. 10). The extrapolation of the linewidths for the vibrational modes at lower frequencies (which emerge using the Fourier interpolation or using models containing more than thousand atoms in the reference cell) is discussed in Sec. *Accounting for anharmonicity at a reduced computational cost*.

The anharmonic linewidths for the 1536(G) and 5184(G) models are determined using the single-valued function Γ_a_[*ω*] shown in Fig. 3 (solid lines).

#### Computational cost

**Fig. 16:**
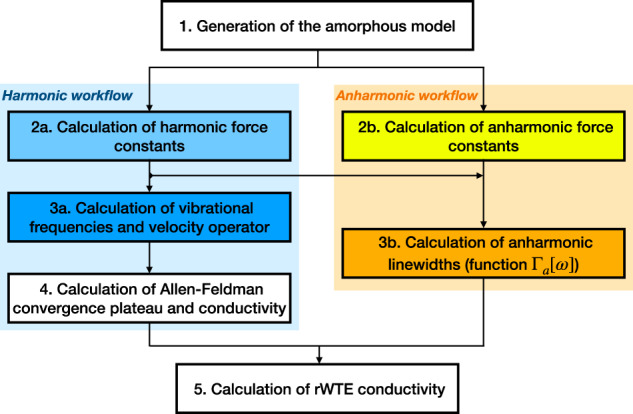
Workflow to compute the bulk limit of Allen-Feldman and rWTE conductivities. The amorphous models employed in this work were generated (step 1) using the melt-quench method (see Sec. *Calculations performed using the GAP potential* for the 1536(G) model, ref. ^60^ for 192(G) and 5184(G), ref. ^63^ for 144(D)) or the bond-switching technique (see ref. ^62^ for 108(D), and ref. ^65^ for 192(D)). After relaxing the atomic position and reference cell to an energy minimum, we computed the harmonic (step 2a) and anharmonic third-order (step 2b) force constants. Then, harmonic force constants were used to compute the vibrational frequencies and velocity operator over a computationally converged **q** mesh (step 3a), thus the convergence plateau for the Allen-Feldman conductivity was determined (see Fig. 2). The broadening *η* determining the beginning of the convergence plateau was used to compute the Allen-Feldman conductivity (step 4). Finally, harmonic and anharmonic force constants were used to compute the linewidths (step 3b), and these were used in combination with the aforementioned broadening *η* to evaluate the rWTE conductivity (step 5, see Eq. (1).

The various steps of the workflow to compute the rWTE conductivity are summarized in Fig. 16, and their computational cost for the different models and simulation methods employed is reported in Table 2.

**Table 2:** Computational cost.

Model	(2a) 2nd order IFC		(3a) velocity operator	(2b) 3rd order IFC		(3b) linewidths
	method (# of displacements)	kCPUh	kCPUh	displacements	kCPUh	kCPUh
108(D)	DFPT 2 × 2 × 2 **q** mesh	12	0.00005 (5 × 5 × 5 **q** mesh)	12348 (3 NN)	9	0.2 (**q** = **0**, 2 cm^−1^)
’	’	’	’	18972 (6 NN)	13	81 (5 × 5 × 5, thm)
’	’	’	’	29592 (10 NN)	21	0.2 (**q** = **0**, 2 cm^−1^)
’	’	’	’	53352 (20 NN)	37	0.2 (**q** = **0**, 2 cm^−1^)
144(D)	DFPT 2 × 2 × 2 **q** mesh	44	0.0001 (5 × 5 × 5 **q** mesh)	26028 (6 NN)	24	0.5 (**q** = **0**, 2 cm^−1^)
192(D)	DFPT 2 × 2 × 2 **q** mesh	102	0.0005 (3 × 3 × 3 **q** mesh)	33300 (6 NN)	50	2.1 (**q** = **0**, 2 cm^−1^)
192(D)	BKS supercell 2 × 2 × 2 (1152)	0.003	0.0005 (3 × 3 × 3 **q** mesh)	32472 (6 NN)	0.1	2.1 (**q** = **0**, 2 cm^−1^)
192(D)	GAP supercell 2 × 2 × 2 (1152)	0.007	0.0005 (3 × 3 × 3 **q** mesh)	33372 (6 NN)	0.2	2.1 (**q** = **0**, 2 cm^−1^)
192(G)	GAP supercell 2 × 2 × 2 (1152)	0.007	0.0005 (3 × 3 × 3 **q** mesh)	34272 (6 NN)	0.2	2.1 (**q** = **0**, 2 cm^−1^)
’	’	’	’	’	’	1.2 (**q** = **0**, 0.4 cm^−1^)
1536(G)	GAP supercell 1 × 1 × 1 (9216)	0.007	0.1 (3 × 3 × 3 **q** mesh)	–	–	–
5184(G)	GAP supercell 1 × 1 × 1 (31104)	1.5	2.1 (3 × 3 × 3 **q** mesh)	–	–	–

**Fig. 17:**
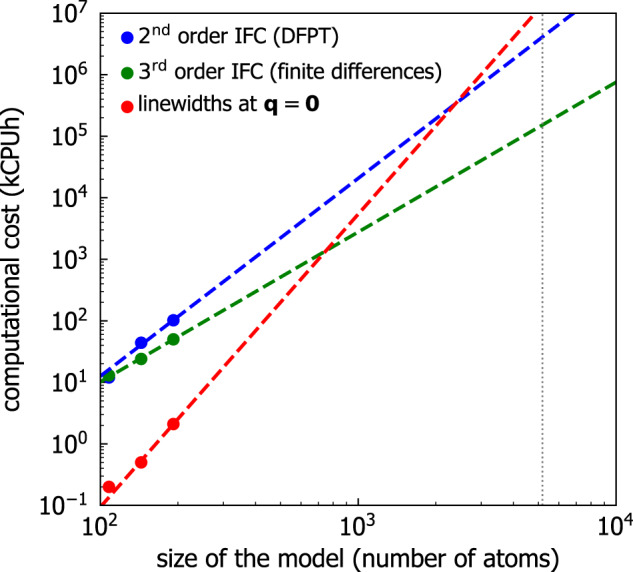
First-principles computational cost as a function of model’s size. Blue is the computational cost for computing second-order IFC using DFPT as implemented in Quantum ESPRESSO^115,118,142^, green is the computational cost for computing third-order IFC using the finite-differences as implemented in ShengBTE^89^ (interfaced with Quantum ESPRESSO), and red is the computational cost for computing anharmonic linewidths at **q** = **0** using Phono3py^91^ and a Gaussian broadening ℏ*σ* = 2 cm^−1^ for the collision operator (with cutoff 3ℏ*σ*). The calculation of the linewidths requires the second- and third-order IFC as inputs, and its computational cost is independent from the technique (DFT or interatomic potential) used to calculate the IFC.

The calculations for the 108(D) and 144(D) models were performed on the Skylake nodes of the SCITAS High Performance Computing facility at the École Polytechnique Fédérale de Lausanne. The calculations for the 192(D), 192(G), 1536(G) and 5184(G) models were performed on the Icelake nodes of the Cambridge Service for Data-Driven Discovery. DFT and DFPT calculations were performed using Quantum ESPRESSO^115,142^, BKS- and GAP-based calculations were performed using LAMMPS^132^ (using the quip^133–135^ interface for GAP). Velocity operator and linewidths were computed using Phono3py^91^. In column 2b, the cutoff used for the third-order force constants (in nearest neighbor units) is reported in parentheses. In column 3b, ‘thm’ refers to the tetrahedron method^91^, 2 cm^−1^ or 0.4 cm^−1^ denote the Gaussian smearing used for the Dirac delta appearing in the linewidth expression. The third-order force constants for the 1536- and 5184-atom models have not been computed, since the subsequent linewidth calculations would have had a prohibitively large computational cost (see Fig. 17). ‘kCPUh’ means 1000 CPU (core) hours.

Figure 4 shows that the rWTE protocol allows to reproduce the thermal conductivity of a large 5184-atom model using a small 192-atom model. As shown in Table 2, atomistic models containing less than 200 atoms have a computational cost that allows to study them by means of standard DFT calculations. Figure 17 shows that the first-principles computational cost rapidly grows with the system size, making it practically impossible to study the large 5184-atom model using DFT. Figure 4 shows that the rWTE protocol allows to determine the above-the-plateau thermal conductivity of *v*-SiO_2_ using a 192-atom model, whose first-principles simulation cost is about 6 orders of magnitude lower than that estimated for simulating from first principles the 5184-atom model.

We also note that once the linewidths at **q** = **0** are used to determine the function Γ_a_[*ω*] through Eqs. (10),(11), the computational cost for evaluating the rWTE conductivity is determined by the size of the **q**-mesh employed. Specifically, for a finite-size model of amorphous material generated in a cubic box, time-reversal symmetry implies that for a *n* × *n* × *n* Gamma-centered **q** mesh the number of independent **q** points^68^ is (*n*^3^ + 1)/2. Once Γ_a_[*ω*] is known, the computationally most expensive part to evaluate the rWTE conductivity is the calculation of the velocity operator at each independent **q** point (as shown by Eqs. (57) and (58) in ref. ^41^, this requires diagonalizing seven dynamical matrices, i.e., at **q**, **q** + *δq*^*β*^, **q** − *δq*^*β*^, with *β* being an index running over the three Cartesian directions). The dynamical matrix has linear size 3 ⋅ *N*_at_, and the formal computational cost for diagonalizing a matrix scales as the third power of its linear size. Table 2 shows that, for all the models analyzed, the computational cost for evaluating the velocity operator on **q** meshes containing less than 100 independent **q**-points is negligible compared to the other parts of the rWTE calculation (namely evaluation of the 2nd and 3rd order force constants, and the calculation of the linewidths at **q** = **0** only to determine Γ_a_[*ω*]).

#### Implementation of the Voigt profile

Given a Gaussian distribution with height $\frac{1}{\pi \eta }$ (i.e., FWHM ${\Delta }_{G}=2\sqrt{\pi \ln 2}\,\eta \approx 2.95\eta$) and a Lorentzian distribution with height $\frac{1}{\pi \gamma }$ (i.e., FWHM Δ_*L*_ = 2*γ*), the Voigt profile is obtained from their convolution, which can be reduced to the following analytical expression^94^:

18 $${{{{\mathcal{F}}}}}_{[\gamma ,\eta ]}(z)=\frac{1}{\pi \eta }K\left[\frac{z}{\eta \sqrt{\pi }},\frac{\gamma }{\eta \sqrt{\pi }}\right],$$

where

19 $$K(x,y)=\frac{y}{\pi }\int\nolimits_{-\infty }^{+\infty }\frac{\exp [-{t}^{2}]}{{y}^{2}+{(x-t)}^{2}}dt.$$

Here we employ the accurate and efficient numerical approximation for the Voigt profile discussed in ref. ^94^. In particular, within such an approximation the Voigt profile is evaluated as:

20 $${{{{\mathcal{F}}}}}_{[\gamma ,\eta ]}(z)=\frac{1}{{\Delta }_{G}+{\Delta }_{L}}{f}_{V}\left(\frac{z}{{\Delta }_{G}+{\Delta }_{L}},\rho \right)$$

where $\rho =\frac{{\Delta }_{L}}{{\Delta }_{G}+{\Delta }_{L}}$, and the numerical function *f*_*V*_ can be written as (in the following we use $\tilde{z}=\frac{z}{{\Delta }_{G}+{\Delta }_{L}}$ to ease the notation):

21 $$\begin{array}{lll}{f}_{V}(\tilde{z},\rho )\,=\,(1-{\eta }_{L}-{\eta }_{I}-{\eta }_{P})\cdot {f}_{G}(\tilde{z},{\gamma }_{G})\\ \qquad\qquad\quad +\,{\eta }_{L}\cdot {f}_{L}(\tilde{z},{\gamma }_{L})+{\eta }_{I}\cdot {f}_{I}(\tilde{z},{\gamma }_{I})+{\eta }_{P}\cdot {f}_{P}(\tilde{z},{\gamma }_{P}).\end{array}$$

Eq. (21) contains the following functions:

22 $${f}_{G}(\tilde{z},{\gamma }_{G})=\frac{1}{\sqrt{\pi }{\gamma }_{G}}\exp (-{\tilde{z}}^{2}/{\gamma }_{G}^{2});$$

23 $${f}_{L}(\tilde{z},{\gamma }_{L})=\frac{1}{\pi }\frac{{\gamma }_{L}}{{\tilde{z}}^{2}+{\gamma }_{L}^{2}};$$

24 $${f}_{I}(\tilde{z},{\gamma }_{I})=\frac{1}{2{\gamma }_{I}}\frac{1}{{[1+{(\tilde{z}/{\gamma }_{I})}^{2}]}^{3/2}};$$

25 $${f}_{P}(\tilde{z},{\gamma }_{I})=\frac{1}{2{\gamma }_{P}}{\left[\frac{2}{\exp (\tilde{z}/{\gamma }_{P})+\exp (-\tilde{z}/{\gamma }_{P})}\right]}^{2}.$$

The parameters *γ*_*G*_, *γ*_*L*_, *γ*_*I*_, *γ*_*P*_ appearing in these functions are polynomials in *ρ*, and depend on Δ_*G*_ + Δ_*L*_:

26 $${\gamma }_{G}=\frac{({\Delta }_{G}+{\Delta }_{L})}{2\sqrt{\ln 2}}\left(1-\rho \mathop{\sum }\limits_{i=0}^{6}{a}_{i}{\rho }^{i}\right);$$

27 $${\gamma }_{L}=\frac{({\Delta }_{G}+{\Delta }_{L})}{2}\left(1-(1-\rho )\mathop{\sum }\limits_{i=0}^{6}{b}_{i}{\rho }^{i}\right);$$

28 $${\gamma }_{I}=\frac{({\Delta }_{G}+{\Delta }_{L})}{2\sqrt{{2}^{2/3}-1}}\mathop{\sum }\limits_{i=0}^{6}{c}_{i}{\rho }^{i};$$

29 $${\gamma }_{P}=\frac{({\Delta }_{G}+{\Delta }_{L})}{2\ln (\sqrt{2}+1)}\mathop{\sum }\limits_{i=0}^{6}{d}_{i}{\rho }^{i}.$$

The coefficients {*a*_*i*_}, {*b*_*i*_}, {*c*_*i*_}, {*d*_*i*_} (*i* = 0,1,...,6) are reported in Table 3.

**Table 3:** Coefficient appearing in the polynomial expansions for the parameters *γ*_*G*_ (Eq. (26)), *γ*_*L*_ (Eq. (27)), *γ*_*I*_ (Eq. (28)), *γ*_*P*_ (Eq. (29)).

*i*	{*a*_*i*_}	{*b*_*i*_}	{*c*_*i*_}	{*d*_*i*_}
0	0.66000	−0.42179	1.19913	1.10186
1	0.15021	−1.25693	1.43021	−0.47745
2	−1.24984	10.30003	−15.36331	−0.68688
3	4.74052	−23.45651	47.06071	2.76622
4	−9.48291	29.14158	−73.61822	−4.55466
5	8.48252	−16.50453	57.92559	4.05475
6	−2.95553	3.19974	−17.80614	−1.26571

The parameters *η*_*L*_, *η*_*I*_, *η*_*P*_ are determined by a similar polynomial expansion:

30 $${\eta }_{L}=\rho \left[1+(1-\rho )\mathop{\sum }\limits_{i=0}^{6}{f}_{i}{\rho }^{i}\right];$$

31 $${\eta }_{I}=\rho (1-\rho )\mathop{\sum }\limits_{i=0}^{6}{g}_{i}{\rho }^{i};$$

32 $${\eta }_{P}=\rho (1-\rho )\mathop{\sum }\limits_{i=0}^{6}{h}_{i}{\rho }^{i}.$$

The coefficients {*f*_*i*_}, {*g*_*i*_}, {*h*_*i*_} (*i* = 0,1,...,6) are reported in Table 4.

**Table 4:** Coefficient appearing in the polynomial expansions for the parameters *η*_*L*_ (Eq. (30)), *η*_*I*_ (Eq. (31)), *η*_*P*_ (Eq. (32)).

*i*	{*f*_*i*_}	{*g*_*i*_}	{*h*_*i*_}
0	−0.30165	0.25437	1.01579
1	−1.38927	−0.14107	1.50429
2	9.31550	3.23653	−9.21815
3	−24.10743	−11.09215	23.59717
4	34.96491	22.10544	−39.71134
5	−21.18862	−24.12407	32.83023
6	3.70290	9.76947	−10.02142

We conclude by noting that, once the linewidths determined from the function detailed in Eqs. (10), (11) are known, evaluating the rWTE conductivity using the numerical scheme for the Voigt profile detailed above does not have a significant impact on the computational cost. More precisely, once frequencies, velocity operators, and linewidths appearing in Eq. (1) are known, evaluating Eq. (1) using the Lorentzian distribution (bare WTE) or the Voigt profile (rWTE) has a computational cost negligible compared to the calculations needed to compute frequencies, velocity operators, and linewidths detailed in Table 2.

## Data Availability

Raw data were generated using the SCITAS High Performance Computing facility at the École Polytechnique Fédérale de Lausanne and the Cambridge Service for Data-Driven Discovery (CSD3). The atomistic models of vitreous silica, *α*-quartz, and *α*-cristobalite studied in this work are available on the Materials Cloud Archive^136,137^.

## Code availability

Quantum ESPRESSO^115^ is available at www.quantum-espresso.org; the scripts related to the computation of the third order force constants using the finite-difference method are available at bitbucket.org/sousaw/thirdorder; Phonopy and Phono3py are available at github.com/phonopy. The GAP potential for silica polymorphs is available in ref. ^60^. LAMMPS^132^ is available at www.lammps.org and the interface for LAMMPS with the GAP potential^133–135^ is available at github.com/libAtoms/QUIP.
